# A spatiotemporal ensemble machine learning framework for generating land use/land cover time-series maps for Europe (2000–2019) based on LUCAS, CORINE and GLAD Landsat

**DOI:** 10.7717/peerj.13573

**Published:** 2022-07-21

**Authors:** Martijn Witjes, Leandro Parente, Chris J. van Diemen, Tomislav Hengl, Martin Landa, Lukáš Brodský, Lena Halounova, Josip Križan, Luka Antonić, Codrina Maria Ilie, Vasile Craciunescu, Milan Kilibarda, Ognjen Antonijević, Luka Glušica

**Affiliations:** 1 OpenGeoHub, Wageningen, The Netherlands; 2 Envirometrix, Wageningen, The Netherlands; 3 Department of Geomatics, Faculty of Civil Engineering, Czech Technical University of Prague, Prague, Czech Republic; 4 MultiOne, Zagreb, Croatia; 5 Terrasigna, Bucharest, Romania; 6 Technical University of Civil Engineering Bucharest, Bucharest, Romania; 7 National Meteorological Administration of Romania, Bucharest, Romania; 8 Department of Geodesy and Geoinformatics, Faculty of Civil Engineering, University of Belgrade, Belgrade, Serbia; 9 GILAB, Belgrade, Serbia

**Keywords:** Landsat, Spatial analysis, Spatiotemporal, Ensemble, Machine learning, Probability, Uncertainty, Land use/land cover, Big data, Environmental monitoring

## Abstract

A spatiotemporal machine learning framework for automated prediction and analysis of long-term Land Use/Land Cover dynamics is presented. The framework includes: (1) harmonization and preprocessing of spatial and spatiotemporal input datasets (GLAD Landsat, NPP/VIIRS) including five million harmonized LUCAS and CORINE Land Cover-derived training samples, (2) model building based on spatial k-fold cross-validation and hyper-parameter optimization, (3) prediction of the most probable class, class probabilities and model variance of predicted probabilities per pixel, (4) LULC change analysis on time-series of produced maps. The spatiotemporal ensemble model consists of a random forest, gradient boosted tree classifier, and an artificial neural network, with a logistic regressor as meta-learner. The results show that the most important variables for mapping LULC in Europe are: seasonal aggregates of Landsat green and near-infrared bands, multiple Landsat-derived spectral indices, long-term surface water probability, and elevation. Spatial cross-validation of the model indicates consistent performance across multiple years with overall accuracy (a weighted F1-score) of 0.49, 0.63, and 0.83 when predicting 43 (level-3), 14 (level-2), and five classes (level-1). Additional experiments show that spatiotemporal models generalize better to unknown years, outperforming single-year models on known-year classification by 2.7% and unknown-year classification by 3.5%. Results of the accuracy assessment using 48,365 independent test samples shows 87% match with the validation points. Results of time-series analysis (time-series of LULC probabilities and NDVI images) suggest forest loss in large parts of Sweden, the Alps, and Scotland. Positive and negative trends in NDVI in general match the land degradation and land restoration classes, with “urbanization” showing the most negative NDVI trend. An advantage of using spatiotemporal ML is that the fitted model can be used to predict LULC in years that were not included in its training dataset, allowing generalization to past and future periods, *e.g.* to predict LULC for years prior to 2000 and beyond 2020. The generated LULC time-series data stack (ODSE-LULC), including the training points, is publicly available via the ODSE Viewer. Functions used to prepare data and run modeling are available via the eumap library for Python.

## Introduction

Anthropogenic land cover change has influenced global climate since the Paleolithic (Kaplan et al., 2011) and continues to be a major driver of regional (Pielke Sr et al., 2002) and global (Houghton et al., 2012) climate change. Furthermore, it is the single largest cause of global biodiversity loss (Sala et al., 2000), and has quantifiable consequences for the availability and quality of natural resources, water, and air (Foley et al., 2005). Key applications of land cover change maps are to inform policy (Duveiller et al., 2020), analyze land-based emissions (Hong et al., 2021), and help estimate local climate extremes (Sy & Quesada, 2020). Quantifying land cover dynamics is often crucial for policy-making at regional and global levels  (Liu et al., 2020b; Trisurat, Shirakawa & Johnston, 2019; Shumba et al., 2020).

Land cover mapping was initially done by visual interpretation of aerial photographs and later on with automated classification of multispectral remotely sensed data with semi-supervised or fully-supervised methods (Townshend et al., 2012; Feranec et al., 2016; Liu et al., 2021). There are currently multiple global (Feng & Bai, 2019; Buchhorn et al., 2020) and regional (Homer et al., 2007; Batista e Silva, Lavalle & Koomen, 2013; Pflugmacher et al., 2019; Malinowski et al., 2020; d’Andrimont et al., 2021) land cover products based on using Machine Learning and offering predictions (or their refinements) at high spatial resolutions for the whole of continental Europe (Table 1). The increasing number of land cover applications and datasets in Europe can largely be attributed to (1) the extensive LUCAS *in-situ* point data being publicly available for research, and (2) NASA’s Landsat and ESA’s Sentinel multispectral images being increasingly available for spatial analysis (Szantoi et al., 2020; Liu et al., 2021).

**Table 1:** Inventory and comparison of existing land cover data products at finer spatial resolutions (≤300 m) available for the continental Europe.

Product/reference	Time span	Spatial resolution	Mapping accuracy	Number of classes	Uncertainty/ probability
CLC	1990, 2000, 2006, 2012, 2018	100 m (25 ha)	≤85%	44	N/N
ESA CCI-LC	1998–2002, 2003–2007, 2008–2012	300-m	73%	22	N/N
Batista e Silva, Lavalle & Koomen (2013)	2006	100-m	70%	42	N/N
S2GLC (Malinowski et al., 2020)	2017	10 m	89%	15	N/N
Pflugmacher et al. (2019)	2014–2016	30 m	75%	12	N/N
GLCFCS30 (Zhang et al., 2020)	2015, 2020	30-m	83%/71%/69%	9/16/24	N/N
Buchhorn et al. (2020)	2015, 2016, 2017, 2018	100 m	80%	10	N/**Y**
ESA WorldCover	2020	10 m	≤75%	≤10	N/N
ELC10 (Venter & Sydenham, 2021)	2020	10 m	90%	8	N/N
ODSE-LULC (our product)	2000, 2001, …, 2019	30 m		43	**Y**/**Y**

However, not all land cover prediction systems perform equally. Vilar et al. (2019) have done extensive evaluation of accuracy of the CLC products for period 2011–2012 using the LUCAS data and found that agreement with LUCAS was slightly higher for CCI-LC (59%; 18 classes) than for CLC (56%; 43 classes). Gao et al. (2020) has evaluated accuracy of the global 30 m resolution products GlobeLand30 with 10 classes (Chen et al., 2015), and GLCFCS30 with 18 classes (Zhang et al., 2020) using the LUCAS point data and concluded that the GlobeLand30-2010 product agrees with LUCAS points up to 89%, while GLCFCS30-2015 agrees up to 85%. The large difference in the agreement reported by Vilar et al. (2019) and Chen et al. (2015) can be attributed to the number of classes in the two studies: the absolute accuracy linearly drops with the number of classes (Herold et al., 2008; Van Thinh et al., 2019), and usually the accuracy results for 6–10 classes *vs* 40 classes can be up to 50% better.

Generally, the accuracy of European land cover mapping projects match those in other parts of the world. For example, Caldern-Loor, Hadjikakou & Bryan (2021) achieved 90% producer’s accuracy when classifying on 6 classes for 7 separate years between 1985 and 2015, using Landsat data of Australia. Tsendbazar et al. (2018) reports similar accuracy levels for Africa. Likewise, Liu et al. (2020a) reports 83% accuracy on 7 classes with 34 years of GLASS data. Finally, the US National Land Cover Database reports accuracy of at least 80% for 16 classes at 30 m in 2001, 2004, 2006, 2008, 2011, 2013, 2016, and 2018 (Homer et al., 2020).

Inglada et al. (2017) report a kappa score of 0.86 for mapping 17 land cover classes for France in 2014. The most-up-to-date land cover products for Europe by Malinowski et al. (2020) report a weighted F1-score of 0.86 based on predicting 13 classes with 2017 Sentinel-2 data. The ESA’s CCI-LC project classified land cover in three multiyear epochs (see Table 1), the last of which achieved an estimated producer’s accuracy of 73% (Arino et al., 2012). Their new WorldCover project (https://esa-worldcover.org/) aims for a consistent accuracy of at least 75% at 10 m spatial resolution. d’Andrimont et al. (2021) recently produced a 10 m resolution European crop type map also by combining LUCAS and plot observations and achieved an overall accuracy of 76% for mapping 19 main crop types for year 2018.

Based on these works, it can be said that the state-of-the-art land cover mapping projects primarily aim at:

(a) Automating the process as much as possible so that land cover maps can be produced almost on monthly or even daily revisit times,
(b) using multi-source Earth Observation data, with especial focus on combining power of the Sentinel-1 and 2 data (Venter & Sydenham, 2021),
(c) producing data of increasingly high spatial and thematic resolution.

Although the modern approaches to land cover mapping listed in Table Table 1 report relatively high levels of accuracy, we recognize several limitations of the general approach:

• Common land cover classification products often only report hard classes, not the underlying probability distributions, limiting the applicability for use cases that would benefit from maximizing either user’s or producer’s accuracy of specific classes in the legend.
• Per-pixel information on the reliability of predictions is often either not reported or not derived at all.
• Many policy makers require time-series land cover data products compatible with legacy products such as CLC and CCI-LC, while most research produces general land cover maps for recent years only.
• Many continental- or global scale land cover mapping missions employ legends with a low number of classes. While achieving high accuracy, such generalized maps are of limited use to large parts of the policy-making and scientific communities.

Land cover data with higher thematic resolution have shown to help improve the performance of subsequent change detection (Buyantuyev & Wu, 2007), as well as the performance and level of detail of modeling land cover trends (Conway, 2009) and other environmental phenomena (Castilla et al., 2009; Zhou et al., 2014). Increasing thematic resolution while limiting the prediction to one trained classifier, however, poses several challenges: (1) training a single model on multi-year data requires extensive data harmonization efforts, and (2) the exponential increase of possible change types with each additional predicted class complicates the manual creation of post-classification temporal consistency rules.

With an increasing spatial resolution and increasing extent of Earth Observation (EO) images, the gap between historic land cover maps and current 10 m resolution products is growing (Van Thinh et al., 2019; d’Andrimont et al., 2021). This makes it difficult to identify key processes of land cover change over large areas (Veldkamp & Lambin, 2001; Vilar et al., 2019). Hence, a balanced and consistent approach is needed that can take into account both accuracy gains due to spatial resolution, and applicability for time-series analysis / change detection for longer periods of time.

The main objective of this paper is to present a framework for spatiotemporal prediction and analysis of LULC dynamics over the span of 20+ years at high thematic resolution, and to assess its usefulness for reproducing the CLC classification system at an annual basis at 30 m resolution. To properly assess the usefulness of the framework, we investigate whether spatiotemporal models (trained on observations from multiple years) generalize better to earth observation data from unknown years than spatial models (trained on observations from a single year). Furthermore, we investigate whether an ensemble machine learning pipeline provides more accurate LULC classifications than single classifiers. Finally, we provide an in-depth analysis of the feasibility to reproduce the CLC classification system by assessing the performance of our framework at various thematic resolution levels.

To this end, we present results of predicting 43 LULC classes from the CLC classification system for continental Europe using spatiotemporal EML at 30 m spatial resolution. These annual predictions are made by a single ensemble model trained on LULC observations ranging from 2000–2018 and a data cube consisting of harmonized annual multispectral Landsat imagery, derived spectral indices, and multiple auxiliary features.

We include the results of multiple accuracy assessments: Firstly, we use 5–fold spatial cross-validation with refitting (Roberts et al., 2017; Lovelace, Nowosad & Muenchow, 2019) to compare the performance of single-year and multi-year models, the performance of the separate component models of our ensemble, and the output of the entire ensemble. Secondly, we test the predictions of our ensemble on the S2GLC validation points, a dataset that was independently collected and published by Malinowski et al. (2020).

We use, as much as possible, a consistent methodology, which implies:

1. Using consistent training data based on consistent sampling methodology and sampling intensity over the complete spacetime cube of interest (LUCAS; d’Andrimont et al. (2020));
2. Using consistent/harmonized Earth Observation images based on the GLAD ARD Landsat product (Potapov et al., 2020), Night Light images NPP/VIIRS (Román et al., 2018) and similar;
3. Providing consistent statistical analysis per every pixel of the space–time cube and per each probability;

Our modeling framework comes at high costs however: The data we have produced is about 50–100 times larger in size than common land cover products with the total size of about 20 TB (Cloud-Optimized GeoTIFFs). A dataset of such volume is more complex to analyze and visualize. To deal with the data size, we ran all processing in a fully automated and fully optimized HPC framework. We refer to the dataset we have produced as ODSE-LULC or short ODSE-LULC.

In the following section we describe how we prepared data, fitted models, tested spatial *vs* spatiotemporal models, and fitted pixel-wise space–time regressions for NDVI and probability time-series. We then report the results and discuss advantages and limitations of spatiotemporal EML, and suggest what we consider could be next development directions and challenges.

## Materials and Methods

### Overview

**Figure 1:**
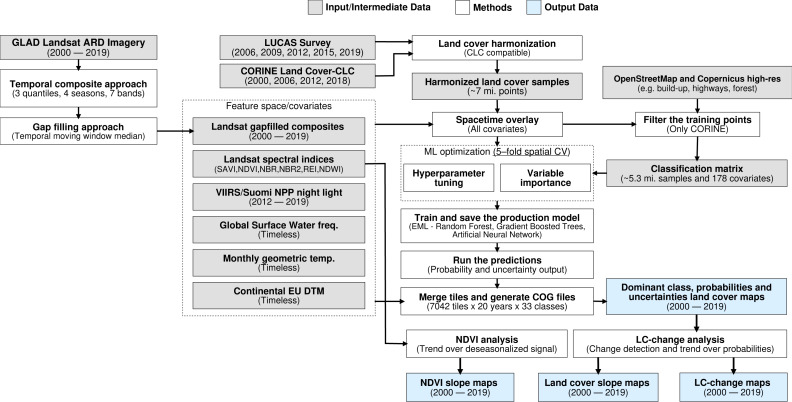
General workflow used to prepare point data and variable layers, fit models and generate annual land cover products (2000–2019). Components of the workflows are described in detail *via* the eumap library (https://eumap.readthedocs.io/), with technical documentation available *via* https://gitlab.com/geoharmonizer_inea.

The annual land cover product for continental Europe was generated using a spatiotemporal modelling approach. This means that all training points are overlaid with EO variables matching both their location and their survey date, so that classification matrix contains spacetime coordinates (*x*, *y*, *t*); then a spatiotemporal model is fitted using the classification matrix. A detailed overview of the workflow used to fit models and produce predictions of land cover is presented in Fig. 1. It was implemented in Python and R programming languages, and is publicly available *via* the eumap library (https://eumap.readthedocs.io/). The eumap library builds upon scikit learn (Pedregosa et al., 2011; Géron, 2019); with “StackingClassifier” as the key function used to produce EML.

All the output predictions were predicted first per tile, then exported as Cloud Optimized Geotiffs (COGs) files and are publicly available through the Open Data Science Europe (ODS-Europe) Viewer, the S3 Cloud Object Service, and from http://doi.org/10.5281/zenodo.4725429. The classification matrix with all training points and variables is available from http://doi.org/10.5281/zenodo.4740691.

### Spatiotemporal ensemble modeling

The annual land cover product for continental Europe was generated with an ensemble of three models and a meta-learner. We used a grid search strategy to find the best hyperparameters and used them to train the final model.

**Figure 2:**
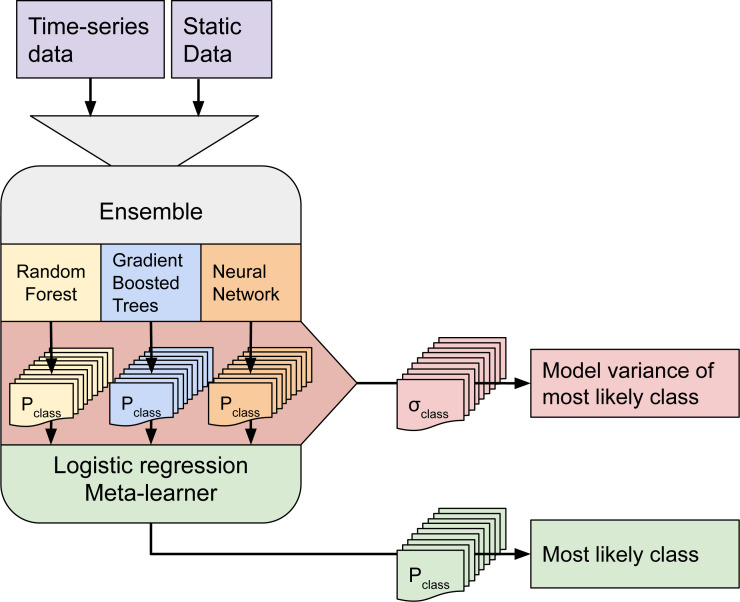
Structure of the ensemble. Time-series data and static data are used to train three component models. Each component model predicts 43 probabilities (one per class). We calculate class-wise uncertainty as a separate output by taking the standard deviation of the three component probabilities per class. The 129 probabilities are used to train the logistic regression meta-learner, which predicts 43 probabilities that are used to map LULC.

Although ensemble training and inference is computationally intensive, it typically achieves higher accuracy than less complex models (Seni & Elder, 2010; Zhang & Ma, 2012). Furthermore, when each component learner predicts a probability per class, it is possible to use the standard deviation of the per-class probabilities as a model-free estimate of the prediction uncertainty (also known as *model variance* (see Fig. 2).

We selected three component learners among an initial pool of 10 learners based on their performance on sample data:

1. Random Forest (Breiman, 2001);
2. Gradient-boosted trees (Chen & Guestrin, 2016);
3. Artificial Neural Network (McCulloch & Pitts, 1943);

Each of these models predicts a probability for each class, resulting in 129 probabilities for 43 classes. These component probabilities are forwarded to the meta-learner, a logistic regression classifier (Defazio, Bach & Lacoste-Julien, 2014), which in turn predicts a single probability per class. The ensemble also outputs the standard deviation of the three component-predicted probabilities per class to generate a class-wise model variance, which can help analyze the data and inform decision-makers where data is more reliable. Because the LUCAS points are based on *in-situ* observations, we considered them as more reliable training data than the CLC centroid points. To prioritize performance on the LUCAS points during model training, we assigned a training weight rating of 100% to the LUCAS points and 85% to the CLC points.

We optimized the hyperparameters of the random forest and gradient boosted trees component learners by minimizing the logistic (log) loss metric (Lovelace, Nowosad & Muenchow, 2019):

(1) \begin{eqnarray*}{L}_{\log \nolimits }(Y,P)=-\log \nolimits \Pr\nolimits (Y{|}P)=- \frac{1}{N} \sum _{i=0}^{N-1}\sum _{k=0}^{K-1}{y}_{i,k}\log \nolimits {p}_{i,k}\end{eqnarray*}

where *Y* is a binary matrix of expected class labels, *N* is the total number of observations, *K* is the number of classes, *P* is the matrix of probabilities predicted by the model, *y*_i,k_ indicates whether sample *i* belongs to class *k*, and *p*_i,k_ indicates the probability of sample *i* belonging to class *j*. A log loss value close to 0 indicate high prediction performance, 0 being a perfect match, while values above 0 indicate progressively worse performance.

We performed 5–fold spatial cross-validation for each different hyperparameter combination (see Table 2. These combinations were generated per model based on a grid search of 5 steps per hyperparameter.

**Table 2:** Minimum and maximum value of each hyperparameter that was optimized for the random forest and gradient boosted tree learners.

Model	Hyperparameter	Lower value	Upper value
Random Forest	Number of estimators	50	100
	Maximum tree depth	5	50
	Maximum number of features	0	0.9
	Minimum samples per leaf	5	30
Gradient boosted trees	Eta	0.001	0.9
	Gamma	0	12
	Alpha	0	1
	Maximum tree depth	2	10
	Number of estimators	10	50

**Figure 3:**
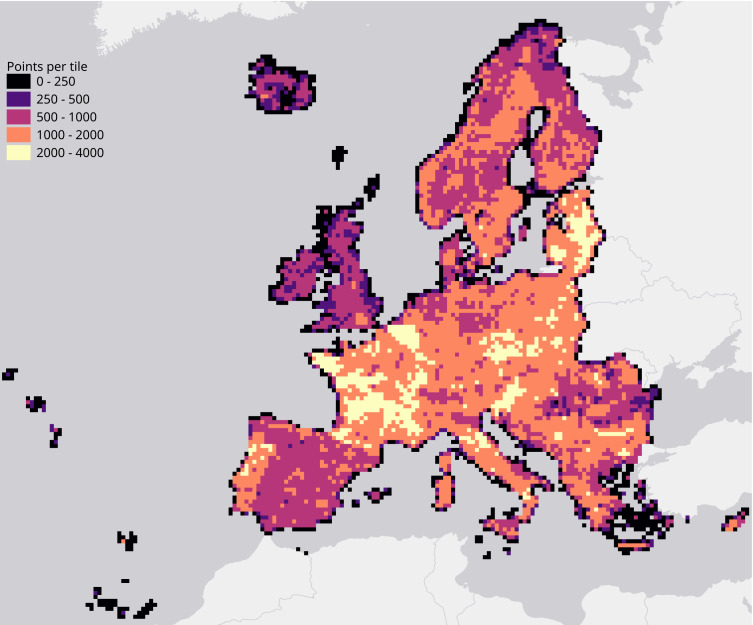
Map of the study area, overlaid with a grid of 30 km tiles that was used for spatial 5-fold cross-validation. Grid color indicates the number of training points aggregated per tile.

We evaluated each set of hyperparameters by performing a spatial 5-fold cross-validation. We did this by creating a Europe-wide grid of 30 km tiles (see Fig. 3) and using the tiles’ unique identifiers to group their overlapping points into 5 folds.

**Table 3:** The ODSE-LULC land cover legend used based on CLC (Bossard et al., 2000). Note: To make table formatting easier, we refer to class 243 as *‘Agriculture with significant natural vegetation‘* in all other tables.

Class name	Class description
111: Continuous urban fabric	Surface area covered for more than 80% by urban structures and other impermeable, artificial features.
112: Discontinuous urban fabric	Surface area covered between 30% and 80% by urban structures and other impermeable, artificial features.
121: Industrial or commercial units	Land units that are under industrial or commercial use or serve for public service facilities.
122: Road and rail networks	Motorways and railways, including associated installations.
123: Port areas	Infrastructure of port areas, including quays, dockyards and marinas.
124: Airports	Airports installations: runways, buildings and associated land.
131: Mineral extraction sites	Areas of open-pit extraction of construction materials (sandpits, quarries) or other minerals (open-cast mines).
132: Dump sites	Public, industrial or mine dump sites.
133: Construction sites	Spaces under construction development, soil or bedrock excavations, earthworks.
141: Urban green	Areas with vegetation within urban fabric.
142: Sport and leisure facilities	Areas used for sports, leisure and recreation purposes.
211: Non-irrigated arable land	Cultivated land parcels under rain-fed agricultural use for annually harvested non-permanent crops, normally under a crop rotation system.
212: Permanently irrigated arable land	Cultivated land parcels under agricultural use for arable crops that are permanently or periodically irrigated.
213: Rice fields	Cultivated land parcels prepared for rice production, consisting of periodically flooded flat surfaces with irrigation channels.
221: Vineyards	Areas planted with vines.
222: Fruit trees and berry plantations	Cultivated parcels planted with fruit trees and shrubs, including nuts, intended for fruit production.
223: Olive groves	Cultivated areas planted with olive trees, including mixed occurrence of vines on the same parcel.
231: Pastures	Meadows with dispersed trees and shrubs occupying up to 50% of surface characterized by rich floristic composition.
241: Annual crops associated with permanent crops	Cultivated land parcels with a mixed coverage of non-permanent (e.g., wheat) and permanent crops (e.g., olive trees).
242: Complex cultivation patterns	Mosaic of small cultivated land parcels with different cultivation types (annual and permanent crops, as well as pastures), potentially with scattered houses or gardens.
243: Land principally occupied by agriculture with significant areas of natural vegetation	Areas principally occupied with agriculture, interspersed with significant semi-natural areas in a mosaic pattern.
244: Agro-forestry areas	Annual crops or grazing land under the wooded cover of forestry species.
311: Broad-leaved forest	Vegetation formation composed principally of trees, including shrub and bush understorey, where broad-leaved species predominate.
312: Coniferous forest	Vegetation formation composed principally of trees, including shrub and bush understorey, where coniferous species predominate.
313: Mixed forest	Vegetation formation composed principally of trees, including shrub and bush understory, where neither broad-leaved nor coniferous species predominate.
321: Natural grasslands	Grasslands under no or moderate human influence. Low productivity grasslands. Often in areas of rough, uneven ground, also with rocky areas, or patches of other (semi-)natural vegetation.
322: Moors and heathland	Vegetation with low and closed cover, dominated by bushes, shrubs (heather, briars, broom, gorse, laburnum *etc*.) and herbaceous plants, forming a climax stage of development.
323: Sclerophyllous vegetation	Bushy sclerophyllous vegetation in a climax stage of development, including maquis, matorral and garrigue.
324: Transitional woodland-shrub	Transitional bushy and herbaceous vegetation with occasional scattered trees. Can represent either woodland degradation or forest regeneration / re-colonization.
331: Beaches, dunes, sands	Natural un-vegetated expanses of sand or pebble/gravel, in coastal or continental locations, like beaches, dunes, gravel pads.
332: Bare rocks	Scree, cliffs, rock outcrops, including areas of active erosion.
333: Sparsely vegetated areas	Areas with sparse vegetation, covering 10–50% of the surface.
334: Burnt areas	Areas affected by recent fires.
335: Glaciers and perpetual snow	Land covered by ice or permanent snowfields.
411 Inland marshes	Low-lying land usually flooded in winter, and with ground more or less saturated by fresh water all year round.
412 Peat bogs	Wetlands with accumulation of considerable amount of decomposed moss (mostly Sphagnum) and vegetation matter. Both natural and exploited peat bogs.
421 Salt marshes	Vegetated low-lying areas in the coastal zone, above the high-tide line, susceptible to flooding by seawater.
422 Salines	Sections of salt marsh exploited for the production of salt by evaporation, active or in process of abandonment, distinguishable from marsh by parcellation or embankment systems.
423 Intertidal flats	Area between the average lowest and highest sea water level at low tide and high tide. Generally non-vegetated expanses of mud, sand or rock lying between high and low water marks.
511: Water courses	Natural or artificial water courses for water drainage channels.
512: Water bodies	Natural or artificial water surfaces covered by standing water most of the year.
521: Coastal lagoons	Str*etc*hes of salt or brackish water in coastal areas which are separated from the sea by a tongue of land or other similar topography.
522: Estuaries	The mouth of a river under tidal influence within which the tide ebbs and flows.

After hyperparameter optimization we trained the three component learners on the full dataset. The meta-learner was trained on the probabilities predicted by each component model during the cross-validation of their optimal hyperparameters.

### Study area and target classification system

The study area covers all countries included in the CLC database, except Turkey (see Fig. 3). The spatiotemporal dataset used in this research contains data from the winter of 1999 to the autumn of 2019.

The target land cover nomenclature was designed based on CLC nomenclature (Bossard et al., 2000) and is available in Table 3. CLC is probably the most comprehensive and detailed European land cover product to date. The CLC program was established in 1985 by the EC to provide geographically harmonized information concerning the environment on the continent. The original CLC dataset is mapped in 44 classes with a minimum mapping unit of 25 ha for areal phenomena and 10 ha for changes. CLC mapping relies on harmonized protocol and guidelines that are shared for country-wise visual photo-interpretation.

The ODSE-LULC nomenclature is identical to the CLC legend, excluding class 523: Sea and ocean, as we omitted such areas from our study area to reduce computation time. The CLC classification system has been reported to be unsuitable for pixel-wise classification due to the inclusion of: (1) heterogeneous and mixed classes defined for polygon mapping (*e.g.*, airports, road and rail networks, complex cultivation patterns, agro-forestry, *etc*.) and (2) classes primarily distinguishable by land use, not land cover (*e.g.*, commercial and industrial units, sports and leisure facilities). We did not remove these classes beforehand to provide objective information about the performance of the CLC level 3 legend for pixel-wise classification, and to enable a complete comparison to the S2GLC nomenclature, which is more optimized for such pixel-based classification.

### Training points

**Figure 4:**
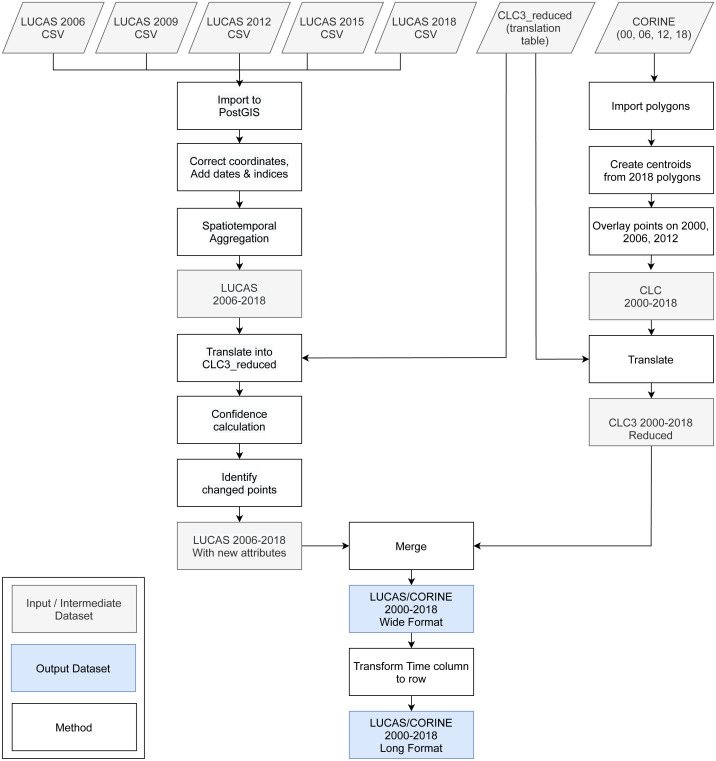
General workflow for merging training points obtained from LUCAS and CLC.

We obtained the training dataset from the geographic location of LUCAS (*in-situ* source) and the centroid of all CLC polygons (as shown in Fig. 4), harmonized according to the 43 land cover classes (see Table 3) and organized by year, where each unique combination of longitude, latitude and year was considered as an independent sample, resulting in more than eight million training points.

**Figure 5:**
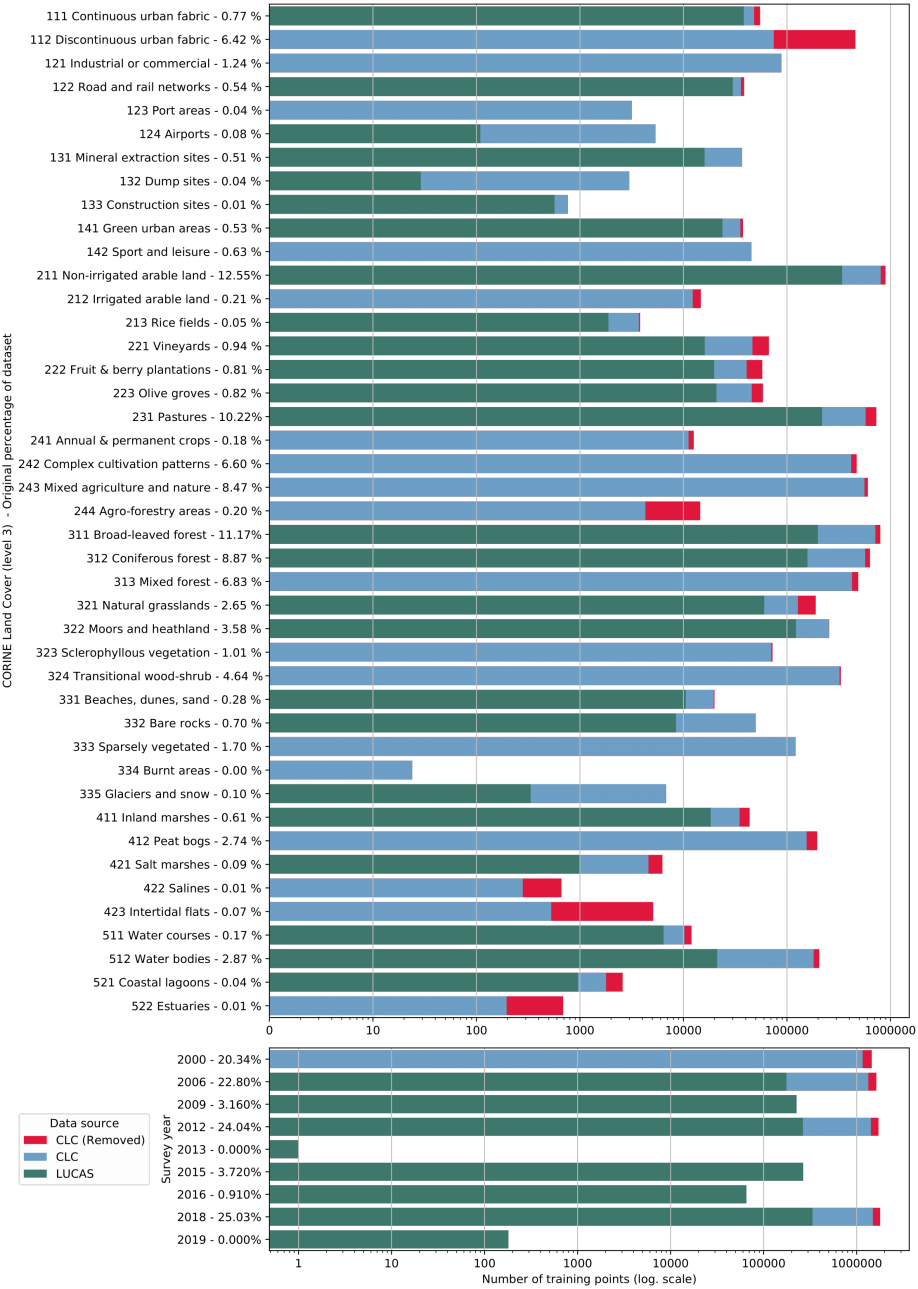
Distribution of training points per data source (blue and green), class (top) and per survey year (bottom). Each bar shows the proportion of points extracted from CLC centroids (blue) and from the LUCAS dataset (green). The proportion of CLC points removed by the OSM and HRL filter step is indicated in red.

The LUCAS data from 2006, 2009, 2012, 2015 and 2018, as provided by Eurostat (obtained from: https://ec.europa.eu/eurostat/web/lucas) is the largest and most comprehensive *in-situ* land cover dataset for Europe. The survey has evolved since 2000 and requires harmonisation before it can be used for mapping over several years. We imported datasets from individual years and harmonized these before merging it into one common database with an automated workflow implemented in Python and SQL (Fig. 1). For the multi-year harmonization procedure we first harmonized attribute names, re-coded variables, harmonized point locations, and aggregated the points based on their location in space and time. After these operations, we translated the LUCAS land cover nomenclature to the ODSE-LULC nomenclature, Table 3, according to the method designed by Buck et al. (2015). The distribution of all reference points per CLC class and per survey year is shown in Fig. 5.

The CLC minimal mapping unit of 25 ha required filtering on the training points before they could be used to represent 30 m resolution LULC, for example, to remove points for *“111: urban fabric”* located in small patches of urban greenery (<25 ha). For this purpose, we extracted vector data from OSM layers for roads, railways, and buildings (obtained from https://download.geofabrik.de/). We then created a 30 m density raster for each feature type. This was done by first creating a 10 m raster where each pixel intersecting a vector feature was assigned the value 100. These pixels were then aggregated to 10 m resolution by calculating the average of every 9 adjacent pixels. This resulted in a 0–100 density layer for the three feature types. Although the digitized building data from OSM offers the highest level of detail, its coverage across Europe is inconsistent. To supplement the building density raster in regions where crowd-sourced OSM building data was unavailable, we combined it with Copernicus We evaluated each set of hyper (obtained from https://land.copernicus.eu/pan-european/high-resolution-layers), filling the non-mapped areas in OSM with the Impervious Built-up 2018 pixel values, which was averaged to 30 m. The probability values produced by the averaged aggregation were integrated in such a way that values between 0–100 refer to OSM (lowest and highest probabilities equal to 0 and 100 respectively), and the values between 101–200 refer to Copernicus HRL (lowest and highest probability equal to 200 and 101 respectively). This resulted in a raster layer where values closer to 100 are more likely to be buildings than values closer to 0 and 200. Structuring the data in this way allows us to select the higher probability building pixels in both products by the single boolean expression: pixel > 50 AND pixel < 150.

We also use HRL products to filter other classes: Table 4 shows the exact conditions points of specific LULC classes needed to meet in order to be retained in our dataset. This procedure is similar to the one used by Inglada et al. (2017). This filtering process removed about 1.3 million points from our training dataset, resulting in a classification matrix with a total of ca.8.1 million samples and 232 variables. The classification matrix used to produce ODSE-LULC is available from http://doi.org/10.5281/zenodo.4740691.

We assessed the quality of the training dataset by comparing it to a number of existing land cover products:

• GLCFCS30–2015 (Zhang et al., 2020);
• GLCFCS30–2020 (Zhang et al., 2020);
• S2GLC (Malinowski et al., 2020);
• The European land cover product for 2015 created by Pflugmacher et al. (2019);
• ELC10 (Venter & Sydenham, 2021).

For each comparison, we reclassified the training dataset to the nomenclature of the target dataset and overlaid all points from our dataset with survey dates from within one year of the land cover product. We then calculated the weighted F1-score as if the points represented predictions. Points with classes of the target products that were completely absent in the training point subsets (due to the target nomenclature of the training points) were removed before these assessments, potentially resulting in varying numbers of classes for the same dataset.

**Table 4:** Per-class conditions applied only to CLC points during the filtering step. All the raster layers were upsampled to 30×30 m resolution by average and the points that did not meet the specified condition were omitted from the training dataset.

		Condition	HRL						OSM		HRL+OSM
Code	Class		Tree cover	Grass	Imp.	Perm. Water	Perm. Wetness	Temp. Wetness	Rails	Roads	Buildings
111	Continuous urban fabric	–									>50 and <150
112	Discontinuous urban fabric										>50 and <150
121	Industrial or commercial units										
122	Road and rail networks and associated land	OR			>30				>30	>30	
123	Port areas										
124	Airports										
131	Mineral extraction sites	AND	= 0	= 0							
132	Dump sites										
133	Construction sites										
141	Green urban areas	( OR ) AND	>0	>0							<50 or >150
142	Sport and leisure facilities										
211	Non-irrigated arable land	AND	= 0						= 0	= 0	<50 or >150
212	Permanently irrigated arable land		= 0						= 0	= 0	<50 or >150
213	Rice fields								= 0	= 0	<50 or >150
221	Vineyards	AND		= 0					= 0	= 0	<50 or >150
222	Fruit trees and berry plantations	AND		= 0					= 0	= 0	<50 or >150
223	Olive groves	AND		= 0					= 0	= 0	<50 or >150
231	Pastures	AND	= 0						= 0	= 0	<50 or >150
241	Annual crops associated with permanent crops								= 0	= 0	<50 or >150
242	Complex cultivation patter								= 0	= 0	<50 or >150
243	Agriculture with significant natural vegetation								= 0	= 0	<50 or >150
244	Agro-forestry areas		>0						= 0	= 0	<50 or >150
311	Broad-leaved forest	AND	>0						= 0	= 0	<50 or >150
312	Coniferous forest	AND	>0						= 0	= 0	<50 or >150
313	Mixed forest		>0						= 0	= 0	<50 or >150
321	Natural grasslands	AND	= 0	>0					= 0	= 0	<50 or >150
322	Moors and heathland								= 0	= 0	<50 or >150
323	Sclerophyllous vegetation								= 0	= 0	<50 or >150
324	Transitional woodland-shrub								= 0	= 0	<50 or >150
331	Beaches, dunes, sand								= 0	= 0	<50 or >150
332	Bare rocks								= 0	= 0	<50 or >150
333	Sparsely vegetated areas								= 0	= 0	<50 or >150
334	Burnt areas								= 0	= 0	<50 or >150
335	Glaciers and perpetual snow								= 0	= 0	<50 or >150
411	Inland marshes	OR					>0	>0	= 0	= 0	<50 or >150
412	Peat bogs								= 0	= 0	<50 or >150
421	Salt marshes								= 0	= 0	<50 or >150
422	Salines								= 0	= 0	<50 or >150
423	Intertidal flats								= 0	= 0	<50 or >150
511	Water courses					>50					
512	Water bodies	–				= 100					
521	Coastal lagoons					>50					
522	Estuaries					>50					

The GLCFCS30 nomenclature was not suitable for direct translation because some land cover types (such as forests) are separated into several subcategories. We therefore aggregated their thematic resolution to the higher level of abstraction described in Zhang et al. (2020). The complete translation scheme is available *via* the GitLab repository of the GeoHarmonizer project (https://gitlab.com/geoharmonizer_inea/spatial-layers).

### Input variables

In this work we combine harmonized time-series data of varying temporal resolution with static datasets. The time-series data consists of the following:

• Seasonal aggregates of Landsat spectral bands (blue, green, red, NIR, SWIR1, SWIR2, thermal), divided into three reflectance quantiles per and four seasons, resulting in 12 layers per band;
• Spectral indices calculated from the seasonal Landsat data: Normalized Difference Vegetation Index (NDVI), Soil Adjusted Vegetation Index (SAVI), Modified Soil Adjusted Vegetation Index (MSAVI), Normalized Difference Moisture Index (NDMI), Landsat Normalized Burn Ratio (NBR), NBR2, REI and Normalized Difference Water Index (NDWI) derived according to formulas in Table 5;
• Terrain Ruggedness Index (TRI) of the Landsat green band (50th reflectance quantile of summer);
• SUOMI NPP VIIRS night light imagery downscaled from 500 m to 30 m resolution (Hillger et al., 2013);
• Monthly geometric minimum and maximum temperature (Kilibarda et al., 2014);

Additional static datasets are:

• Probability of surface water occurrence at 30 m resolution pekel2016high;
• Continental EU DTM-based elevation and slope in percent (Hengl et al., 2021);

All variables used by our model are derived from remotely sensed EO data from multiple sources, the largest share being derived from Landsat imagery. Although EO data with higher spatial and temporal resolution, as well as actual surface reflection values are available (*e.g.*, Sentinel-2), such sources do not cover the timespan required for the long-term analysis proposed by this framework. The Landsat data used in this work was obtained by downloading the Landsat ARD, provided by GLAD (Potapov et al., 2020), for the years 1999 to 2019 and for the entire extent of continental Europe (see eumap landmask (Hengl et al., 2021)). This imagery archive was screened to remove the cloud and cloud shadow pixels, maintaining only the quality assessment-QA values labeled as clear-sky according to GLAD. Second, we averaged the individual images by season according to three different quantiles (25th, 50th and 75th) and the following calendar dates for all periods:

• Winter: December 2 of previous year until March 20 of current year;
• Spring: March 21 until June 24 of current year;
• Summer: June 25 until September 12 of current year;
• Fall: September 13 until December 1 of current year.

We decided to use the equal length definition provided by Trenberth (1983) to represent four seasons and matching the beginning and end of each season with the 16-day intervals used by Potapov et al. (2020). From more than 73 TB of input data we produced 84 images (3 quantiles × 4 seasons × 7 Landsat bands) for each year with different occurrences of no-data values due to cloud contamination in all observations of a specific season.

**Table 5:** Spectral indices derived from the Landsat data and used as additional variables in the spatiotemporal EML.

Spectral index	Equation	Reference
NDVI	$ \frac{nir-red}{nir+red} $	Tucker (1979)
SAVI	$ \frac{nir-red}{(nir+red+0.5)\times 1.5} $	Huete (1988)
MSAVI	$ \frac{(2\times nir+1)-\sqrt{(2\times nir+1)^{2}-8\times (nir-red)}}{2} $	Qi et al. (1994)
NDWI	$ \frac{green-swir2}{green+swir2} $	Gao (1996)
NBR	$ \frac{nir-thermal}{nir+thermal} $	Key & Benson (1999)
NDMI	$ \frac{nir-swir1}{nir+swir1} $	Jin & Sader (2005)
NBR2	$ \frac{swir1-thermal}{swir1+thermal} $	Key & Benson (2006)
REI	$ \frac{nir-blue}{nir+blue} \times nir$	Shahi et al. (2015)

We next impute all missing values in the Landsat temporal composites using the *“Temporal Moving Window Median”* TMWM algorithm, implemented in python and publicly available in the eumap library (see Fig. 1). The algorithm uses the median values derived from temporal neighbours to impute a missing value using pixels from (1) the same season, (2) neighboring seasons and 3 the full year. For example, for a missing value in the spring season, the algorithm first tries to use values from spring seasons of neighbouring years. If no pixel value is available for the entire period (*i.e.,* 2000–2019), the algorithm tries to use values from winter and summer of neighbouring years. If no pixel value is available from data of adjacent seasons from the same year, pixel values from adjacent years are used to derive the median values. Ultimately, a missing value will not receive an impute value only if the pixel lacks data throughout the entire time-series. The median calculation considers different sizes of temporal windows, which expands progressively for each impute attempt (*i.e.,* time_win_size parameter); in this work we used a maximum time_win_size of 7. We selected the TMWM approach from a set of 4 algorithms through a benchmarking process. To our knowledge, it provides the best combination of gap-filling accuracy and computational costs on the scale of this project.

We include several spectral indices as a form of feature engineering because they are each designed and tested to help identify or distinguish different types of land cover. Table Table 5 provides an overview of how we derived them from the Landsat data. This was done for each quantile and each season, resulting in 4 × 3 = 12 variables per spectral index.

The TRI (Riley, DeGloria & Elliot, 1999) gives an indication of how different pixel values are from those of its neighbors. Is usually calculated from elevation data, but we include it as a derivative of the Landsat green band in order to help the model distinguish between pixels that are part of larger, homogeneous regions from pixels that are located inside more heterogeneous landscapes (*e.g.*, airports, urban green areas, and forest edges).

The Suomi-NPP VIIRS night light imagery (Hillger et al., 2013) was included to introduce a variable that may help the model recognize the built-up environment, but also distinguish different types of land use within that category. This data is originally in 500 m resolution, but we re-sampled them to 30 m using a cubic spline.

The geometric minimum and maximum temperature is a geometric transformation of latitude and the day of the year (Kilibarda et al., 2014). We include these variables to improve performance on LULC classes that occur in different situations under distant latitudes e.g.coniferous forest in Greece and Norway. It can be defined anywhere on the globe using Eq. (2):

(2) \begin{eqnarray*}{t}_{min}& =24.2\cdot \cos \nolimits \phi -15.7\cdot (1-\cos \nolimits \theta )\cdot \sin \nolimits {|}\phi {|}-0.6\cdot \frac{z}{100} \end{eqnarray*}

(3) \begin{eqnarray*}{t}_{max}& =37\cdot \cos \nolimits \phi -15.4\cdot (1-\cos \nolimits \theta )\cdot \sin \nolimits {|}\phi {|}-0.6\cdot \frac{z}{100} \end{eqnarray*}

where *θ* is derived as:

(4) \begin{eqnarray*}\theta =(day-18)\cdot \frac{2\pi }{365} +{2}^{1-\mathrm{sgn}(\phi )}\cdot \pi .\end{eqnarray*}

where *day* is the day of year, *ϕ* is the latitude, the number 18 represents the coldest day in the northern and warmest day in the southern hemisphere, *z* is the elevation in meter, 0.6 is the vertical temperature gradient per 100 m, and sgn denotes the signum function that extracts the sign of a real number.

We include a long-term (35-year) probability estimate of surface water occurrence pekel2016high based on the expectation that it would improve model performance when classifying LULC classes associated with water, such as wetlands and rice fields.

### Accuracy assessment

We evaluate the suitability of the proposed framework with three assessments:

1. Comparison of spatial and spatiotemporal models;
2. 5-fold spatial cross-validation;
3. Validation on S2GLC point data.

We compare the performance of spatial and spatiotemporal models to assess whether training models on data from multiple years can improve their ability to generalize to data from unknown years. We expect models trained on observations from multiple years to generalize better on data from unknown years than models trained on observations from a single year. In order to investigate this, we trained multiple ensemble models on several subsets of our training data that were selected from either one or several years, and validated them on data from years included in their training data and on observations from 2018, the last year of the training dataset, upon which no model was trained.

The validation on the S2GLC point data is included to assess the extent to which the choice of legend affects the classification accuracy of our framework. The S2GLC legend contains less classes and does not

The results produced by the 5-fold spatial cross-validation are used to assess four characteristics of the proposed methodology:

1. The difference in performance between the ensemble model and its component models;
2. classification accuracy of the framework when reproducing the 43-class CLC classification system;
3. consistency of prediction accuracy by the framework through time;
4. consistency of prediction accuracy by the framework through space;

In all comparisons and experiments, we discriminate model performance with the Weighted F1-score metric (Van Rijsbergen, 1980):

(5) \begin{eqnarray*}{\mathrm{WF}}_{1}=\sum _{c=1}^{n}{S}_{c}\cdot \frac{2\cdot {P}_{c}\cdot {R}_{c}}{{P}_{c}+{R}_{c}} \end{eqnarray*}

where *n* is the number of classes, and *S*_*c*_ is the support (the number of training points), *P*_*c*_ the precision (producer’s accuracy), and *R*_*c*_ the recall (user’s accuracy) of a given class *c*. We used a weighted version of this metric because it distinguishes classification performance more strictly on imbalanced datasets, such as the one used in this work.

#### Spatial cross-validation

Before mapping LULC in continental Europe for all years, we performed spatial 5-fold cross-validation using the hyperparameters of the final EML model to assess its performance. The predictions for the points from each left-out fold were merged into one set of predicted values, which we used to assess the performance of our final model. We did this for each of the three levels in the CLC nomenclature (with 43, 15, and 5 classes) to investigate the effect of legend size. We aggregated predictions to the higher level in the hierarchy by taking the highest probability among subclasses within the same higher level class before selecting the most probable class. Besides this general performance on the total dataset, we also analyzed the performance of the ensemble per class, year, and cross-validation tile.

Analyzing the performance per class and per level in the hierarchy allows us to quantify the performance increase gained from aggregating specific classes. We do this by calculating the weighted average of the F1-score of all sub-classes of a higher-level class (*e.g.*, 311: Broad-leaved forest, 312: Coniferous forest, and 313: Mixed forest, which together comprise the level 2 class 31: Forests and seminatural areas). Finally, we subtract the weighted average F1-score of the subclasses from the F1-score of the higher-level class to quantify the performance gain. This value will tend to be higher when the model frequently confuses sub-classes of a higher-level class, as aggregation then removes more classification errors.

We analyzed the temporal and spatial consistency of our model performance by calculating the weighted F1-scores for the cross-validation predictions on points from each separate year and tile, respectively. We calculated the standard deviation of these scores to assess the consistency of the model.

Finally, we also compare the cross validation log loss score per class, as well as aggregated per CLC level, with a baseline log loss score. This baseline log loss is what a random classifier would score when predicting on a given dataset. A dataset with more classes and a more unequal distribution has a higher baseline log loss score. We also calculate a log loss ratio to give a measure of model performance that is agnostic of the number and distribution of classes, instead only reflecting how well a given model performed given the difficulty of its task. We define this ratio as follows:

(6) \begin{eqnarray*}R(Y,P)=1- \frac{{L}_{\log \nolimits }(Y,P)}{{B}_{\log \nolimits }(Y,P)} \end{eqnarray*}

where *L*_log_ indicates the log loss score of the prediction and *B*_log_ indicates the baseline log loss score that would be scored by a randomly predicting model. A ratio of 0 means that the model did not outperform a random predictor, a ratio of 1 means a perfect prediction with a log loss score of 0.

#### Validation on S2GLC points

After training an ensemble model with the same hyperparameters on all training data, we classified LULC in 2017. This prediction was validated with the S2GLC dataset which Malinowski et al. (2020) used to validate their 2017 land cover product. The dataset contains 51,926 points with human-verified land cover classifications which were collected with a stratified random sampling method from 55 proportionally selected regions of Europe.

As the S2GLC points follow a different nomenclature, we translated the ODSE-LULC predicted classes according to Table 6. Because any predicted classes outside the S2GLC nomenclature (labeled as 000: None in Table 6) would be automatically counted as errors, we performed two validations: (1) a conservative assessment that included points with such predictions, and (2) an optimistic assessment where they were omitted.

**Table 6:** Reclassification key used to validate the predictions of our ensemble model on the S2GLC point dataset collected by Malinowski et al. (2020).

S2GLC	ODSE-LULC
	111: Continuous urban fabric
	112: Discontinuous urban fabric
	121: Industrial or commercial units
111: Artificial Surfaces	122: Road and rail networks and associated land
	123: Port areas
	124: Airports
	132: Dump sites
	133: Construction sites
311: Broadleaf tree Cover	311: Broad-leaved forest
312: Coniferous Tree Cover	312: Coniferous forest
211: Cultivated Areas	211: Non-irrigated arable land
	212: Permanently irrigated arable land
	213: Rice fields
	241: Annual crops associated with permanent crops
	242: Complex cultivation patterns
	243: Agriculture with significant natural vegetation
	244: Agro-forestry areas
231: Herbaceous Vegetation	231: Pastures
	321: Natural grasslands
411: Marshes	411: Inland Marshes
	421: Salt Marshes
	422: Salines
	423: Intertidal Flats
322: Moors and Heathland	322: Moors and heathland
331: Natural Material Surfaces	131: Mineral extraction sites
	331: Beaches, dunes, sands
	332: Bare rocks
000: None	141: Green urban areas
	142: Sport and leisure facilities
	222: Fruit trees and berry plantations
	223: Olive groves
	313: Mixed Forest
	324: Transitional woodland-shrub
	333: Sparsely vegetated areas
	334: Burnt areas
412: Peat Bogs	412: Peat Bogs
335: Permanent Snow	335: Glaciers and perpetual snow
323: Sclerophyllous Vegetation	323: Sclerophyllous vegetation
221: Vineyards	221: Vineyards
511: Water Bodies	511: Water courses
	512: Water bodies
	521: Coastal lagoons
	522: Estuaries

#### Comparison of ensemble and component models

Previous studies have shown that ensemble models can outperform their component models (Seni & Elder, 2010; Zhang & Ma, 2012). To investigate if this was the case for our approach, we compared the spatial cross-validation accuracy of the three selected component models with that of the full ensemble. We also compared variable importance of the gradient boosted trees and random forest models in order to discover to what extent the different models used different parts of the available feature space.

#### Comparison of spatial and spatiotemporal models

We decided to use a spatiotemporal model trained on reference data from multiple years because we expect it to generalize better to data from years that were not included in its training data. We expect this because the EO covariates are more diverse in multi-year datasets, which leads to a larger feature space and likely reduces overfitting.

We also expected better performance from spatiotemporal models because combining data from multiple years allows for larger training datasets, which generally improves the predictive power of a model.

To investigate these two benefits, we trained three types of models:

1. Spatial models, trained on 100,000 points from a single year;
2. small spatiotemporal models, trained on 100,000 points sampled from our multi-year dataset;
3. large spatiotemporal models, trained on 100,000 points from each year of our multi-year dataset.

We trained a small and a large spatiotemporal model to gain separate insight into the effects of dataset size and dataset diversity. The years 2000, 2006, 2009 and 2012 had sufficient points for this experiment, resulting in 4 spatial models, 1 small spatiotemporal model, and 1 large spatiotemporal model. We then evaluated each model’s classification performance on a dataset sampled from the same years as the model’s training data, and a dataset sampled from 2018, which was excluded from the training data selection. Every model’s validation dataset was $ \frac{1}{3} $^rd^ the size of its training dataset. The validation on data from 2018 represents each model’s ability to generalize to data from years that it was not trained to classify. We averaged the performance of all spatial models to obtain the performance of one *’spatial model’*.

To investigate the effect of combining the CLC and LUCAS points, we performed this experiment three times by training and validating on only CLC points, only LUCAS points, and a combination of CLC and LUCAS points.

### Time-series analysis

After classifying LULC in Europe between 2000–2019, we analyzed the dynamics of land cover predicted by our model in three ways:

1. Probability and NDVI trend analysis using logistic regression on NDVI and the probabilities for key classes;
2. change class per year and between 2001–2018;
3. prevalent change mapping.

These LULC change dynamics were not validated and serve as a means of analyzing the output of the presented framework. Furthermore, the GLAD ARD data-set by Potapov et al. (2020) is produced for analyzing land cover change but should not be used for land surface reflectance applications directly. Therefore we do not use NDVI trends as an indication of absolute vegetation vigor but only as a relative measure of change. Also, NDVI trends are only applied as a tool to understand the changes and to enhance interpretation.

**Figure 6:**
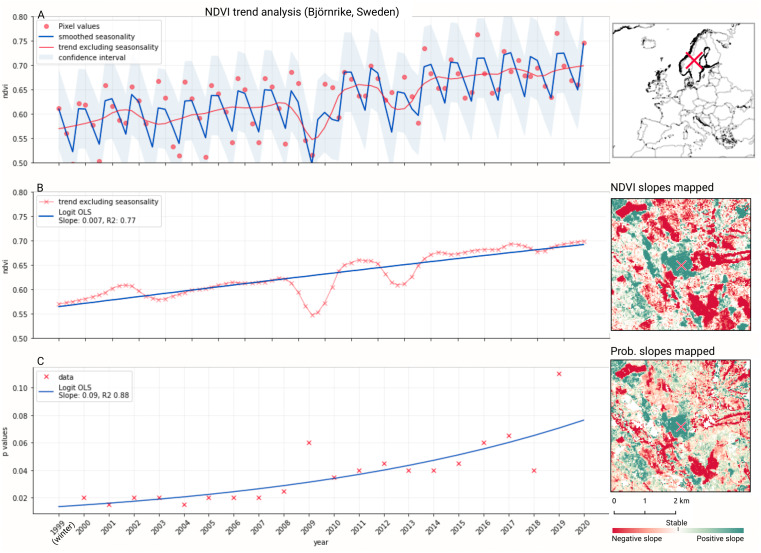
Example of deseasonalization (Seabold & Perktold, 2010) and subsequent Logit OLS applied on a single pixel in Sweden (Coordinates: 62° 24′43. 7”N 13° 56′00.3”E): (A) red dots represent pixel values, the blue line represents a local weighted regression smoothed line based on the pixel values plus a light blue area indicating the confidence interval, the red line represents the trend after removing the seasonal signal; (B) red line and crosses represent the trend after removing the seasonal signal, the blue line visualizes the regression model based NDVI values in the logit space; (C) Trend analysis on probability values for non-irrigated arable land. In the case above the gradient value is 0.09 with the model R-square =0.88.

We analyzed the trend over the years between 2000 and 2019 by fitting an OLS regression model on the time-series of probabilities of every pixel. We use the coefficient as a proxy for the gradual change through time. Because probabilities only have meaningful values between 0 and 1 and NDVI are only meaningful for values between −1 and 1, we applied a logit transformation to the input data of the OLS analysis. We applied this trend analysis on the four most prevalent LULC classes: (1) coniferous forest, (2) non-irrigated arable land, (3) broad leaved forest, and (4) pastures. We also applied this method on a deseasonalized (Seabold & Perktold, 2010) NDVI time-series (see Fig. 6 and present this trend analysis as an additional tool to qualitatively appraise large-scale, long-term trends.

In order to visualise change implied by our LULC predictions, we first implement a smoothing post-processing strategy before categorizing change processes. The smoothing strategy considers the classification of a pixel in the previous and next years. If a pixel is classified as one class, but as another single class in the year before and after, this classification is considered an error. In such a case, the pixel’s class is changed to match the previous and subsequent class. We call this a *“T-3 temporal filter“*.

After this preprocessing step, we categorize LULC change processes by applying the change classes seen in the Copernicus land cover map (Buchhorn et al., 2020) to our classification scheme. We translated the CLC classes to the land cover classes used by the Copernicus land cover map according to Table 7. Some examples of changes include: changing from Dump sites into Urban fabric is classified as *“No change”*, changing from Non-irrigated arable land into Urban fabric to *“Urbanization”*, changing from Airports to Mineral extraction sites to *“Other” etc*. Two notable exceptions are the *“forest loss”* and *“Reforestation”* classes. In this paper we will refer to *“Forest loss”* and *“Forest increase”* instead. We renamed these change classes because we wanted to avoid making assumptions regarding the drivers of the detected trends in forest cover.

**Table 7:** Harmonization scheme used to convert ODSE-LULC nomenclature to Copernicus Global Land Cover classes. On the left side, ODSE-LULC classes are converted to Forest, Other Vegetation, Wetland, Bare, Cropland, Urban, and Water classes. Each transition from one Copernicus class to another is then categorized into a change class in the cross-table.

**ODSE-LULC class**	**Copernicus change class**	**Forest**	**Other vegetation**	**Wetland**	**Bare**	**Cropland**	**Urban**	**Water**
311: Broad-leaved forest	**Forest**		Forest loss			Deforestation and crop expansion	Deforestation and urbanization	Water expansion
312: Coniferous forest						
321: Natural grasslands	**Other Vegetation**	Reforestation		Other	Desertification	Crop expansion	Urbanization	
322: Moors and heathland								
324: Transitional woodland-shrub								
323: Sclerophyllous vegetation								
411: Inland wetlands	**Wetland**		Wetland degradation		Wetland degradation and desertification	Wetland degradation and crop expansion	Wetland degradation and urbanization	
421: Maritime wetlands								
332: Bare rocks	**Bare**		Other			Crop expansion	Urbanization	
333: Sparsely vegetated areas							
334: Burnt areas							
335: Glaciers and perpetual snow							
335: Beaches, dunes, and sands							
211: Non-irrigated arable land	**Cropland**		Land abandonment		Land abandonment and desertification			
212: Permanently irrigated arable land							
213: Rice fields							
221: Vineyards							
222: Fruit trees and berry plantations							
223: Olive groves							
231: Pastures							
111: Urban fabric	**Urban**		Other					
122: Road and rail networks and associated land					
123: Port areas					
124: Airports					
131: Mineral extraction sites					
132: Dump sites					
133: Construction sites					
141: Green urban areas					
511: Water courses	**Water**	Water reduction						
512: Water bodies			
523: Sea and ocean			
522: Estuaries			
521: Coastal lagoons			

In order to identify and visualize the dominant LULC change trends in Europe, we mapped the *“prevalent change“* at two scales of aggregation: 5 × 5 km and 20 × 20 km. We created a Europe-covering grid with cells at both scales. Then, we counted the number of 30 × 30 m pixels of each change class within each grid cell. The predominant change class (see Table Table 7) was then assigned to each grid cell. We also calculated *“change intensity”* by dividing the number of 30 × 30 m pixels of the prevalent change class, by the sum of all pixels in each grid cell. For example, at a 20 × 20 km scale, each grid cell contains have (20, 000/30)⋅(20, 000/30) = 444, 444 pixels. If the prevalent change class is present in >94,000 pixels this means that it covers >20% of the total area.

## Results

### Quality of reference data

Table 8 shows how well each compared land cover product matched ODSE-LULC training data. The comparison with S2GLC with our points from 2016 and 2018 resulted in the highest F1-scores, while the land cover product made by Pflugmacher et al. (2019) fits more closely to the 2015 subset (0.657). The 2019 point subset was considered too small to perform any meaningful comparison between ELC10 and GLCFCS30. The number of classes can vary per dataset per year because we excluded all classes from the translated dataset that do not appear in the target land cover product.

**Table 8:** Weighted F1-score of other land cover products when validated with the ODSE-LULC training dataset.

Land cover product	Validation year	Data source	Samples	Weighted F1-Score	Number of classes	Res. (m)
S2GLC	2016	LUCAS	756	0.724	8	10
Pflugmacher et al. (2019)	2016	LUCAS	719	0.719	10	30
GLCFCS30–2015	2016	LUCAS	724	0.677	10	30
Pflugmacher et al. (2019)	2015	LUCAS	144,027	0.657	11	30
S2GLC	2018	LUCAS	295,152	0.653	11	10
S2GLC	2018	CLC	1,000,063	0.604	12	10
ELC10	2018	LUCAS	42,629	0.596	8	10
GLCFCS30–2015	2015	LUCAS	138,342	0.503	12	30
ELC10	2018	CLC	172,382	0.456	8	10
GLCFCS30–2020	2018	LUCAS	308,838	0.424	12	30
GLCFCS30–2020	2018	CLC	1,026,914	0.420	12	30

### Spatiotemporal ensemble modelling results

The EML model optimization resulted in the following hyperparameters and architecture:

• Random forest: Number of trees equal to 85, maximum depth per tree equal to 25, number of variables to find the best split equal to 89, and 20 as minimum number of samples per leaf.
• Gradient boosted trees: Number of boosting rounds equal to 28, maximum depth per tree equal to 7, minimum loss reduction necessary to split a leaf node equal to 1, L1 regularization term on weights equal to 0.483, learning rate equal to 0.281, greedy histogram algorithm to construct the trees, and softmax as objective function.
• Artificial Neural Network: Four fully connected hidden layers with 64 artificial neurons each; ReLU as activation function, dropout rate equal to 0.15 and batch normalization in all the layers; softmax as activation function for output layer; batch size and number of epochs equal to 64 and 50, respectively; and Adam with Nesterov momentum as optimizer considering 5e−4 as learning rate.
• Logistic Regression: SAGA solver and multinomial function to minimize the loss.

**Figure 7:**
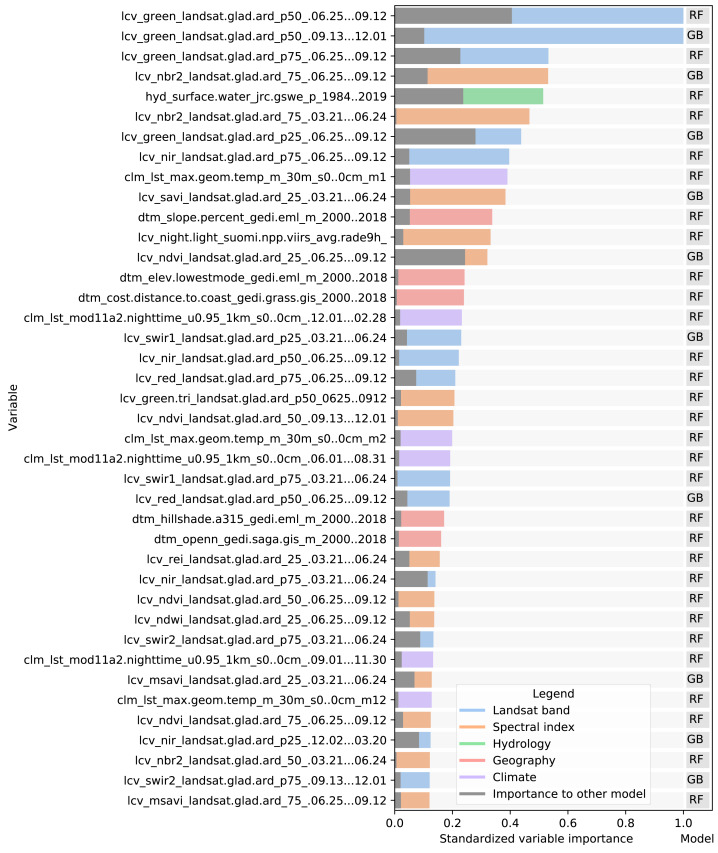
Standardized importance of the top-40 most important variables to the random forest and gradient boosted tree models. The colored bar indicates the highest importance of the variable among the two models. This model is indicated to the right of each bar. The corresponding grey bar indicates the importance to the other model. The color of each bar indicates the data type. Each variable name is prefixed with either LCV (either part of a Landsat band or a landsat-derived spectral index), HYD (Hydrological data), CLM (climatic data), or DTM (digital terrain model). This prefix is followed by the specific data source, *e.g.*, [color or index]_landsat indicates a Landsat band or derived spectral index. The last part of each name indicates the timespan over which the data was aggregated.

The variable importance, generated by the two tree-based learners and presented in Fig. 7, shows that the 50th quantile for summer and winter of the Landsat green band were most important to the random forest and gradient boosted tree models, respectively. In addition to spectral bands, several Landsat-derived spectral indices (NBR2, SAVI, NDVI, REI, NDWI, MSAVI) appear amongst the 40 most important variables. Global surface water frequency was the third most important for the random forest. Figure 7 also shows that the summer aggregates of Landsat green (25th quantile) and NDVI are the two most important variables where the highest importance among the two models is less than double the importance of the other model. Except for Landsat green and NDVI, most variables were found important by only one model. For instance, the geometric temperatures and nighttime land surface temperatures were only important for the random forest. The differences in variable importance indicate that the component models use different parts of the feature space before their predictions are combined by the meta-learner, suggesting that ensembles can utilize a wider proportion of the feature space than single models.

### Accuracy assessment results

#### Spatial cross-validation

We performed 5-fold spatial cross-validation with the final hyperparameters for our ensemble. The predictions on the left-out folds were aggregated to assess model performance on the entire dataset. Table 9 shows that the model achieved higher weighted user and producer accuracy, as well as F1-score and log loss ratio, when predictions were aggregated to their next level in the CLC hierarchy. Table 10 shows that the model only achieved an F1-score over 0.5 for 10 out of 43 classes (112, 121, 211, 213, 311, 312, 332, 335, 412, 512). The model performed best when predicting 512: Water bodies (0.924), 335: Glaciers and perpetual snow (0.834), and 412: Peat bogs (0.707). It achieved the lowest F1-scores for 334: Burnt areas (0.011), 132: Dump sites (0.026) and 133: Construction sites (0.065). However, log loss ratios for each class and each CLC level overall were higher than 0, indicating that the model assigned probabilities more accurately than a random classifier even for the most difficult classes.

When the predictions were aggregated to 14 level 2 classes (see Table 11), the model performed best when classifying 51: Inland waters (0.924), 31: Forests and seminatural areas (0.813) and 41: Inland wetlands (0.708). The biggest increase in performance through aggregation to level 2 was in 31: Forests, as the weighted average F1-score of its subclasses (311,312,313) was 0.553. The least accurately predicted classes were 14: Artificial, non-agricultural vegetated areas (0.308), 13: Mine, dump and construction sites (0.370) and 22: Permanent crops (0.412).

**Table 9:** Producer’s and user’s accuracy, Weighted F1-score, and Log loss of the ensemble predictions during spatial cross-validation.

Corine level	Number of classes	Prod acc.	User acc.	Weighted F1	Log loss	Baseline log loss	Log loss ratio
1	5	0.835	0.835	0.834	0.456	2.018	0.774
2	14	0.636	0.639	0.509	1.033	3.596	0.713
3	43	0.494	0.502	0.491	1.544	5.142	0.700

**Table 10:** Classification report for 43 CLC level 3 classes, based on the predictions made with 5-fold spatial cross-validation.

CLC code (level 3)	Producer Acc.	User Acc.	F1-score	Support	Log loss	Baseline log loss	Log loss ratio
111: Continuous urban fabric	0.523	0.166	0.252	51,989	0.0230	0.0388	0.409
112: Discontinuous urban fabric	0.509	0.572	0.539	92,151	0.0256	0.0623	0.590
121: Industrial or commercial units	0.496	0.623	0.552	129,661	0.0382	0.0821	0.535
122: Road and rail networks and associated land	0.294	0.068	0.111	39,832	0.0244	0.0311	0.213
123: Port areas	0.543	0.321	0.403	3,994	0.0018	0.0042	0.578
124: Airports	0.300	0.023	0.043	6,702	0.0049	0.0067	0.265
131: Mineral extraction sites	0.482	0.307	0.375	53,447	0.0264	0.0397	0.335
132: Dump sites	0.375	0.013	0.026	6,509	0.0048	0.0065	0.267
133: Construction sites	0.217	0.038	0.065	6,728	0.0047	0.0067	0.299
141: Green urban areas	0.312	0.125	0.179	15,717	0.0091	0.0141	0.350
142: Sport and leisure facilities	0.407	0.200	0.268	64,308	0.0326	0.0463	0.297
211: Non-irrigated arable land	0.604	0.733	0.662	998,381	0.1892	0.3735	0.493
212: Permanently irrigated arable land	0.447	0.146	0.221	29,786	0.0139	0.0243	0.428
213: Rice fields	0.762	0.496	0.601	4,839	0.0020	0.0050	0.596
221: Vineyards	0.506	0.308	0.383	66,213	0.0287	0.0474	0.394
222: Fruit trees and berry plantations	0.411	0.131	0.199	63,659	0.0344	0.0459	0.251
223: Olive groves	0.432	0.355	0.390	63,578	0.0244	0.0459	0.469
231: Pastures	0.455	0.529	0.489	529,466	0.1509	0.2415	0.375
241: Annual crops associated with permanent crops	0.269	0.067	0.107	16,883	0.0101	0.0150	0.326
242: Complex cultivation patter	0.348	0.351	0.349	594,648	0.1942	0.2624	0.260
243: Agriculture with significant natural vegetation	0.355	0.373	0.363	782,237	0.2558	0.3176	0.194
244: Agro-forestry areas	0.276	0.052	0.087	10,497	0.0060	0.0099	0.396
311: Broad-leaved forest	0.537	0.660	0.592	855,499	0.1971	0.3373	0.416
312: Coniferous forest	0.596	0.646	0.620	759,215	0.1644	0.3112	0.472
313: Mixed forest	0.461	0.377	0.414	612,430	0.1707	0.2680	0.363
321: Natural grasslands	0.406	0.314	0.354	400,875	0.1431	0.1971	0.274
322: Moors and heathland	0.493	0.350	0.409	301,693	0.1100	0.1591	0.309
323: Sclerophyllous vegetation	0.311	0.372	0.339	143,521	0.0532	0.0890	0.403
324: Transitional woodland-shrub	0.472	0.431	0.450	724,404	0.2117	0.3013	0.297
331: Beaches, dunes, sand	0.551	0.207	0.301	25,688	0.0147	0.0214	0.312
332: Bare rocks	0.664	0.495	0.567	58,234	0.0162	0.0427	0.621
333: Sparsely vegetated areas	0.522	0.471	0.495	152,571	0.0457	0.0935	0.511
334: Burnt areas	0.224	0.006	0.011	2,263	0.0021	0.0026	0.177
335: Glaciers and perpetual snow	0.852	0.818	0.834	7,250	0.0008	0.0072	0.883
411: Inland marshes	0.425	0.228	0.297	39,784	0.0192	0.0310	0.382
412: Peat bogs	0.684	0.731	0.707	174,314	0.0333	0.1039	0.680
421: Salt marshes	0.505	0.441	0.471	5,598	0.0023	0.0057	0.600
422: Salines	0.481	0.081	0.139	320	0.0002	0.0004	0.577
423: Intertidal flats	0.497	0.209	0.295	788	0.0004	0.0010	0.570
511: Water courses	0.360	0.108	0.166	11,214	0.0068	0.0105	0.353
512: Water bodies	0.895	0.956	0.924	187,981	0.0108	0.1103	0.902
521: Coastal lagoons	0.594	0.429	0.498	1,904	0.0006	0.0022	0.708
522: Estuaries	0.382	0.082	0.135	353	0.0002	0.0005	0.566
Macro average	0.460	0.327	0.356	8097140	0.083	0.137	0.452
Weighted average	0.494	0.502	0.491		0.157	0.253	0.389
Accuracy	0.502			
Kappa score	0.459			
Log Loss (baseline)	1.544 (5.142)			

**Table 11:** Classification report for 14 CLC level 2 classes, based on the predictions made with 5-fold spatial cross-validation.

CLC code (level 2)	Producer Acc.	User Acc.	f1-score	Support	Log loss	Baseline log loss	Log loss ratio
11: Urban Fabric	0.643	0.535	0.584	144,140	0.039	0.089	0.564
12: Industrial, commercial and transport units	0.568	0.551	0.559	180,189	0.057	0.107	0.469
13: Mine, dump and construction sites	0.533	0.283	0.370	66,684	0.032	0.048	0.331
14: Artificial, non-agricultural vegetated areas	0.479	0.227	0.308	80,025	0.038	0.055	0.315
21: Arable land	0.622	0.738	0.675	1,033,006	0.191	0.382	0.500
22: Permanent crops	0.558	0.326	0.412	193,450	0.072	0.113	0.363
23: Pastures	0.455	0.529	0.489	529,466	0.151	0.242	0.375
24: Heterogeneous agricultural areas	0.488	0.496	0.492	1,404,265	0.364	0.461	0.212
31: Forests and seminatural areas	0.788	0.840	0.813	2,227,144	0.302	0.588	0.487
32: Shrub and/or herbaceous vegetation associations	0.592	0.511	0.548	1,570,493	0.384	0.492	0.218
33: Open spaces with little or no vegetation	0.736	0.591	0.656	246,006	0.061	0.136	0.555
41: Inland wetlands	0.719	0.697	0.708	214,098	0.044	0.122	0.643
42: Coastal wetlands	0.591	0.465	0.520	6,706	0.003	0.007	0.618
51: Inland waters	0.913	0.936	0.924	199,195	0.013	0.115	0.884
52: Marine waters	0.614	0.392	0.479	2,273	0.001	0.003	0.699
Macro average	0.620	0.541	0.569	8,097,140	0.117	0.197	0.482
Weighted average	0.636	0.639	0.634		0.262	0.420	0.393
Accuracy	0.639			
Kappa score	0.565			
Log Loss (baseline)	1.033 (3.596)			

**Table 12:** Classification report for 5 CLC level 1 classes, based on the predictions made with 5-fold spatial cross-validation.

CLC code (level 1)	Producer Acc.	User Acc.	F1-score	Support	Log loss	Baseline log loss	Log loss ratio
1: Artificial surfaces	0.784	0.613	0.688	471,038	0.123	0.222	0.445
2: Agricultural areas	0.798	0.854	0.825	3,160,187	0.457	0.669	0.317
3: Forest and seminatural areas	0.872	0.848	0.860	4,043,643	0.526	0.693	0.241
4: Wetlands	0.722	0.696	0.708	220,804	0.045	0.125	0.639
5: Water bodies	0.917	0.936	0.926	201,468	0.013	0.116	0.884
Macro average	0.819	0.789	0.802	8,097,140	0.233	0.365	0.505
Weighted average	0.835	0.835	0.834		0.450	0.626	0.309
Accuracy	0.835			
Kappa score	0.720			
Log Loss (baseline)	0.456 (2.018)			

Table 12 shows that at the highest level of aggregation with 5 general classes, the model classified 5: Water bodies most accurately (0.926) and 1: Artificial surfaces the least (0.688). The best performance improvement from aggregation was for 2: Agricultural areas, as the weighted average F1-score of its subclasses (21, 22, 23, 24) was 0.546, but increased with 0.279 upon aggregation.

**Figure 8:**
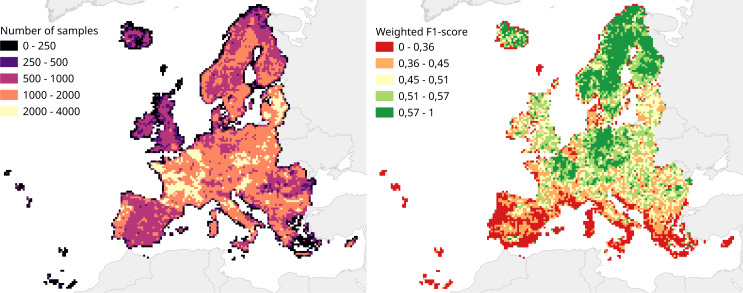
Comparison of number of samples and cross-validation performance. Both metrics are visualized for each tile in the 30 km tiling system used for spatial cross-validation. Left: Number of samples per tile. Right: Weighted F1-score per tile.

**Figure 9:**
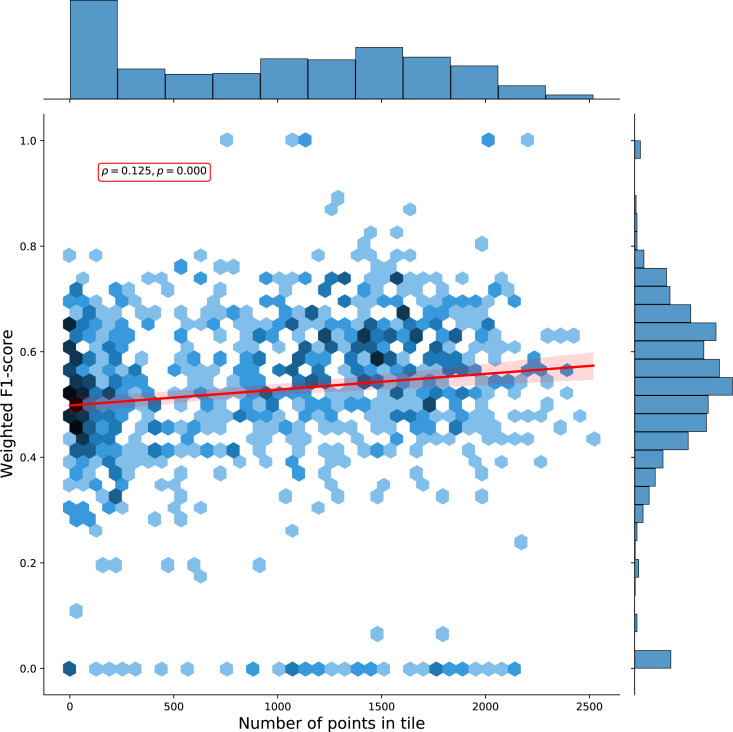
Hexbin plot of the weighted F1-score and number of overlapping points per tile. The Pearson correlation coefficient of 0.125 (p: 0.000) indicates there is a weak positive correlation between the number of points in a tile and the cross-validation weighted F1-score.

We calculated a separate weighted F1-score for each tile that was used for spatial cross-validation to investigate spatial patterns in classification performance. The average weighted F1-score per tile was 0.463, with a standard deviation of 0.150. Figure 8 shows a disparity in performance between northern and southern Europe. Figure 9 shows that there is a significant correlation (0.125, *p* = 0.000) between the number of reference points and the weighted F1 score of a tile.

We calculated a separate weighted F1-score for all cross-validation predictions from each separate year. Table 13 shows that the average weighted F1-score per year was 0.489 with a standard deviation of 0.135. It only scored higher than 0.5 on years with less than 1 million points.

#### Validation on S2GLC points

We validated the ensemble on S2GLC dataset. We overlaid 49,897 S2GLC points with our input variables for 2017 and classified 43 LULC classes with our model. These 43-class predictions were reclassified to the S2GLC nomenclature. 3,484 points had a predicted class that was not in the S2GLC nomenclature (see Table 6). The *‘conservative’* assessment (on all 49,897 points) including the non-S2GLC classes resulted in a weighted F1-score of 0.854 and a kappa score of 0.794 (see Table 14). The *‘optimistic‘* assessment excluding non-S2GLC predictions resulted in a weighted F1-score of 0.889 and a kappa score of 0.867 (see Table 15).

Taking into account possible noise from the translation process, these results are similar to those reported by Malinowski et al. (2020). Weighted average user and producer accuracy and F1-scores are also higher than our cross-validation scores at all thematic resolution levels (see Table 9). They are also higher than what we obtained when we transformed our cross-validation predictions to the S2GLC nomenclature, which yielded a weighted F1-score 0.611 and a kappa score of 0.535.

**Figure 10:**
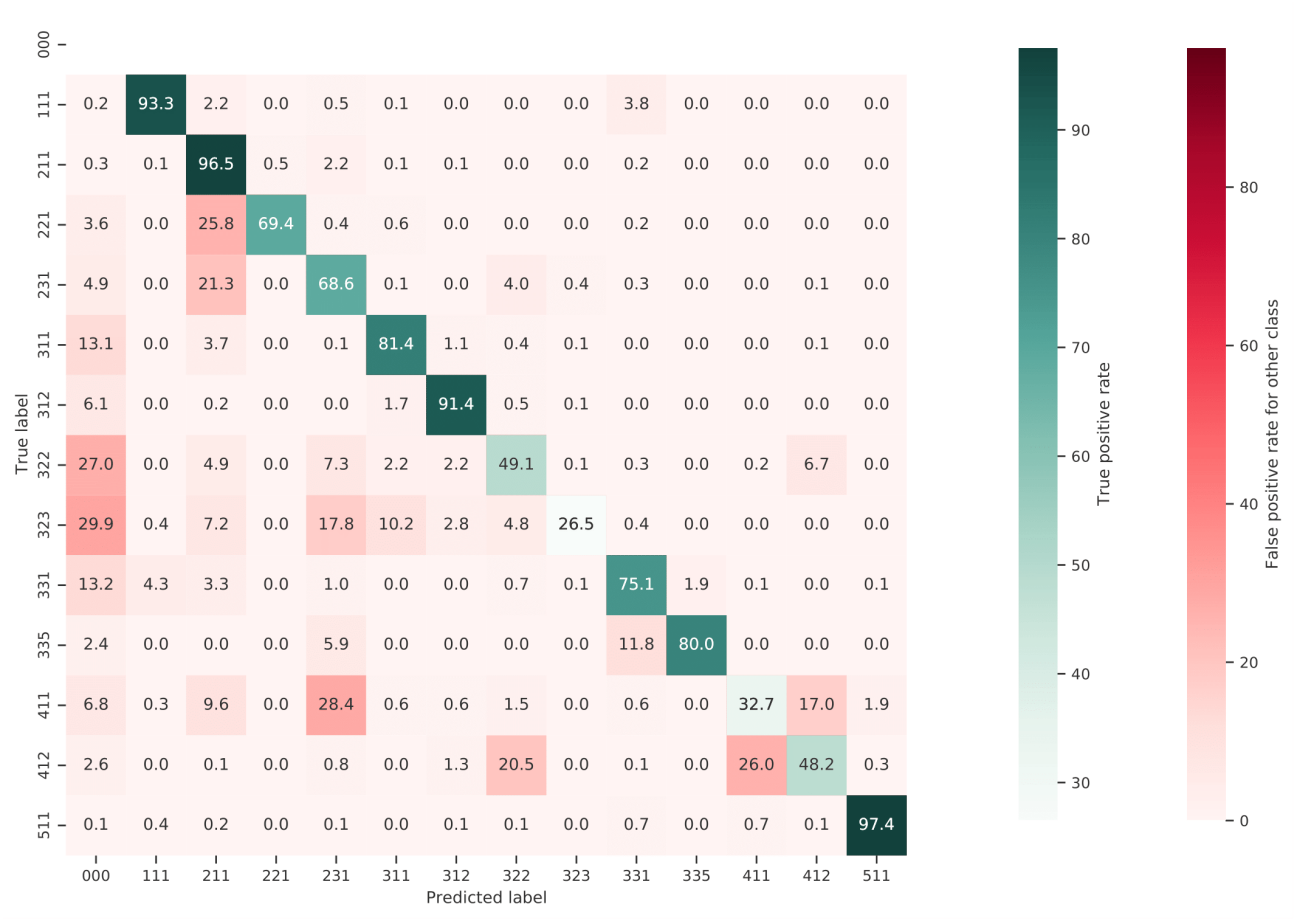
Normalized confusion matrix of our prediction on the independently collected S2GLC validation points. Each cell shows the percentage of the true label predicted as the predicted label.

**Figure 11:**
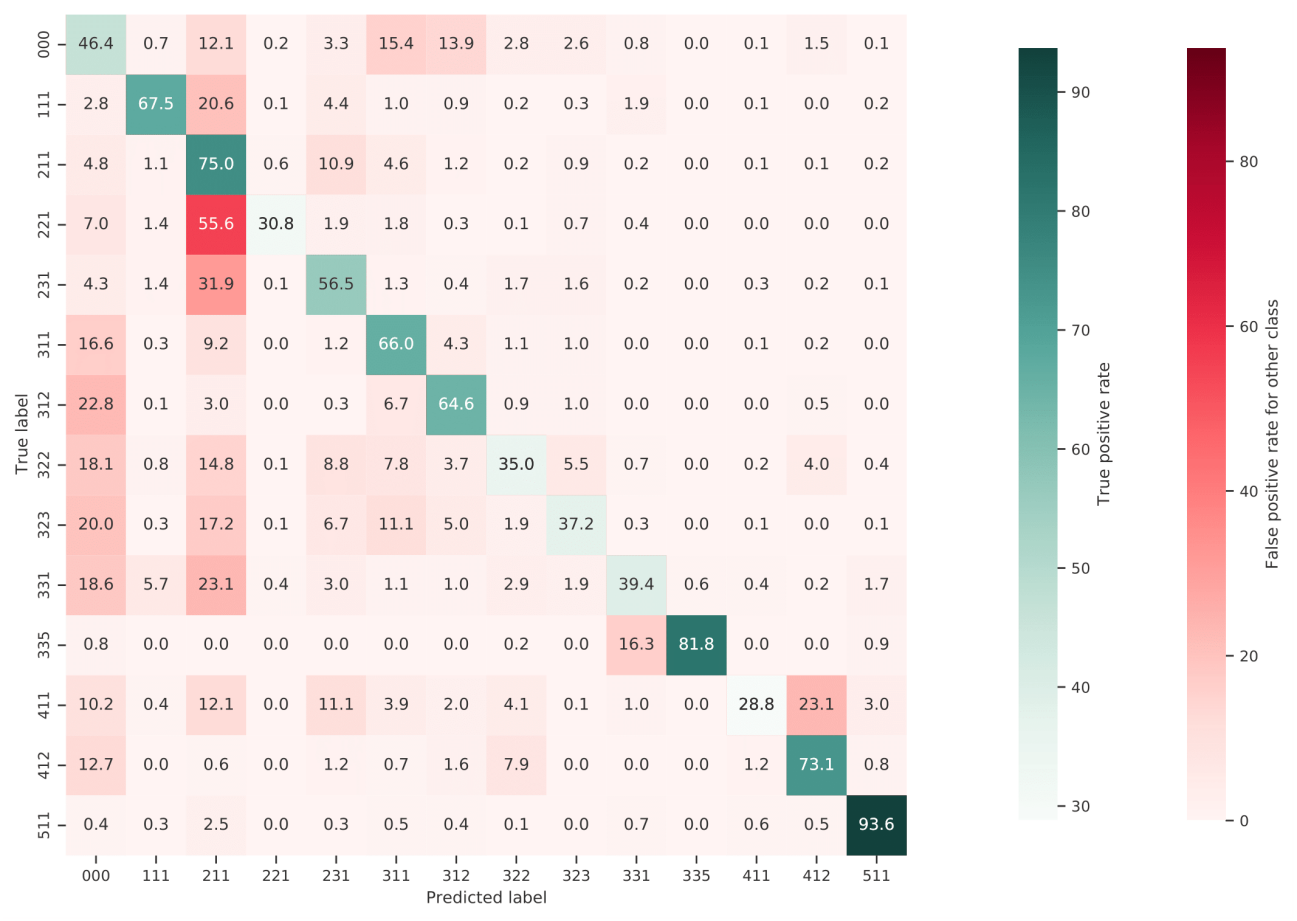
Normalized confusion matrix of the predictions made by our model during spatial cross-validation on our own dataset, reclassed to the S2GLC nomenclature. Each cell shows the percentage of the true label predicted as the predicted label.

Figure 10 shows a normalized confusion matrix of our validation on the S2GLC dataset. It shows the rate at which each true class (rows) was predicted as each other class (columns). The diagonal cells report the true positive rate of each class. Class 000 represents classes not present in the S2GLC dataset; as there were no ground truth points in the dataset with these classes, the top row of the matrix is empty. The matrix shows that, when normalized for support, the biggest sources of error were the incorrect classification of classes 323: Sclerophyllous vegetation and 322: Moors and Heathland as classes not in the S2GLC dataset with 29.9% and 27.0% of all errors for these classes, respectively, and of 411: Marshes as 231: Herbaceous vegetation (28.4%). We include a similar confusion matrix of our cross-validation predictions (Fig. 11, transformed to the S2GLC nomenclature, to allow a comparison between our cross-validation and independent validation. It shows that many classes have a higher true positive rate in the independent validation on S2GLC points than in our cross-validation results, except for 211: Cultivated areas, 335: Permanent snow cover, and 412: Peatbogs.

#### Comparison of spatial and spatiotemporal models

We trained two types of models and compared their performance: Spatial models, which were trained on 100,000 points sampled from one year, and spatiotemporal models, which were trained on 100,000 points equally distributed across multiple years. Table 16 shows the weighted F1-scores obtained through validating each model on 33,333 points from the same year(s) as its training data, and on 33,333 points from the year 2018, which was left out of all training datasets.

**Table 13:** Cross-validation performance of our ensemble model per year.

Year	Weighted F1-score	Support
2000	0.497	1,658,715
2006	0.491	1,852,645
2009	0.558	225,416
2012	0.487	1,971,812
2015	0.588	265,830
2016	0.632	65,235
2018	0.481	2,057,306
2019	0.535	180
Average	0.489	1,012,142
Standard deviation	0.135	882,783

**Table 14:** Conservative classification report of our 2017 LULC prediction on 49,897 S2GLC points that counts 3484 points with predicted classes without an equivalent S2GLC class as errors (141: Green urban areas, 142: Sport and leisure facilities, 222: Fruit trees and berry plantations, 223: Olive groves, 313: Mixed forest, 324: Transitional woodland-shrub, 333: Sparsely vegetated areas, and 334: Burnt areas).

S2GLC class	Producer Acc.	User Acc.	F1-score	Support
111: Artificial surfaces	0.933	0.933	0.933	1,826
211: Cultivated areas	0.849	0.965	0.903	13,470
221: Vineyards	0.826	0.694	0.754	500
231: Herbaceous vegetation	0.861	0.686	0.764	6,776
311: Broadleaf tree cover	0.967	0.814	0.884	10,944
312: Coniferous tree cover	0.975	0.914	0.943	8,626
322: Moors and heathland	0.641	0.491	0.556	2,070
323: Sclerophyllous vegetation	0.780	0.265	0.396	815
331: Natural material surfaces	0.915	0.751	0.825	2,110
335: Permanent snow cover	0.624	0.800	0.701	85
411: Marshes	0.331	0.327	0.329	324
412: Peatbogs	0.629	0.482	0.546	745
511: Water bodies	0.992	0.974	0.983	1,606
Macro average	0.737	0.650	0.680	49,897
Weighted average	0.892	0.830	0.854	
Accuracy	0.830			
Kappa score	0.794			

**Table 15:** Optimistic classification report of our 2017 LULC prediction on 49,897 S2GLC points where all 3484 points with predicted classes without an equivalent S2GLC class were removed before calculating accuracy metrics (141: Green urban areas, 142: Sport and leisure facilities, 222: Fruit trees and berry plantations, 223: Olive groves, 313: Mixed forest, 324: Transitional woodland-shrub, 333: Sparsely vegetated areas, and 334: Burnt areas).

S2GLC class	Producer Acc.	User Acc.	F1-score	Support
111: Artificial surfaces	0.933	0.935	0.934	1,823
211: Cultivated areas	0.849	0.967	0.905	13,429
221: Vineyards	0.826	0.720	0.769	482
231: Herbaceous vegetation	0.861	0.722	0.785	6,441
311: Broadleaf tree cover	0.967	0.937	0.952	9,512
312: Coniferous tree cover	0.975	0.973	0.974	8,098
322: Moors and heathland	0.641	0.672	0.656	1,511
323: Sclerophyllous vegetation	0.780	0.378	0.509	571
331: Natural material surfaces	0.915	0.866	0.889	1,831
335: Permanent snow cover	0.624	0.819	0.708	83
411: Marshes	0.331	0.351	0.341	302
412: Peatbogs	0.629	0.494	0.554	726
511: Water bodies	0.992	0.975	0.984	1,604
Macro average	0.794	0.755	0.766	46,413
Weighted average	0.893	0.892	0.889	
Accuracy	0.892			
Kappa score	0.867			

The results show that all models performed better when validated on points from the same year as their training data, regardless of data source. However, spatial models achieved higher F1-scores on average when trained and validated on only LUCAS points, while the spatiotemporal models performed better when trained and validated on only CLC points.

The spatiotemporal model trained on only CLC points achieved the highest F1-scores for both known-year and unknown-year classification. This model outperformed spatial models on known-year classification by 2.7% and unknown-year classification by 3.5% as seen in Table 16.

#### Comparison of ensemble and component models

**Figure 12:**
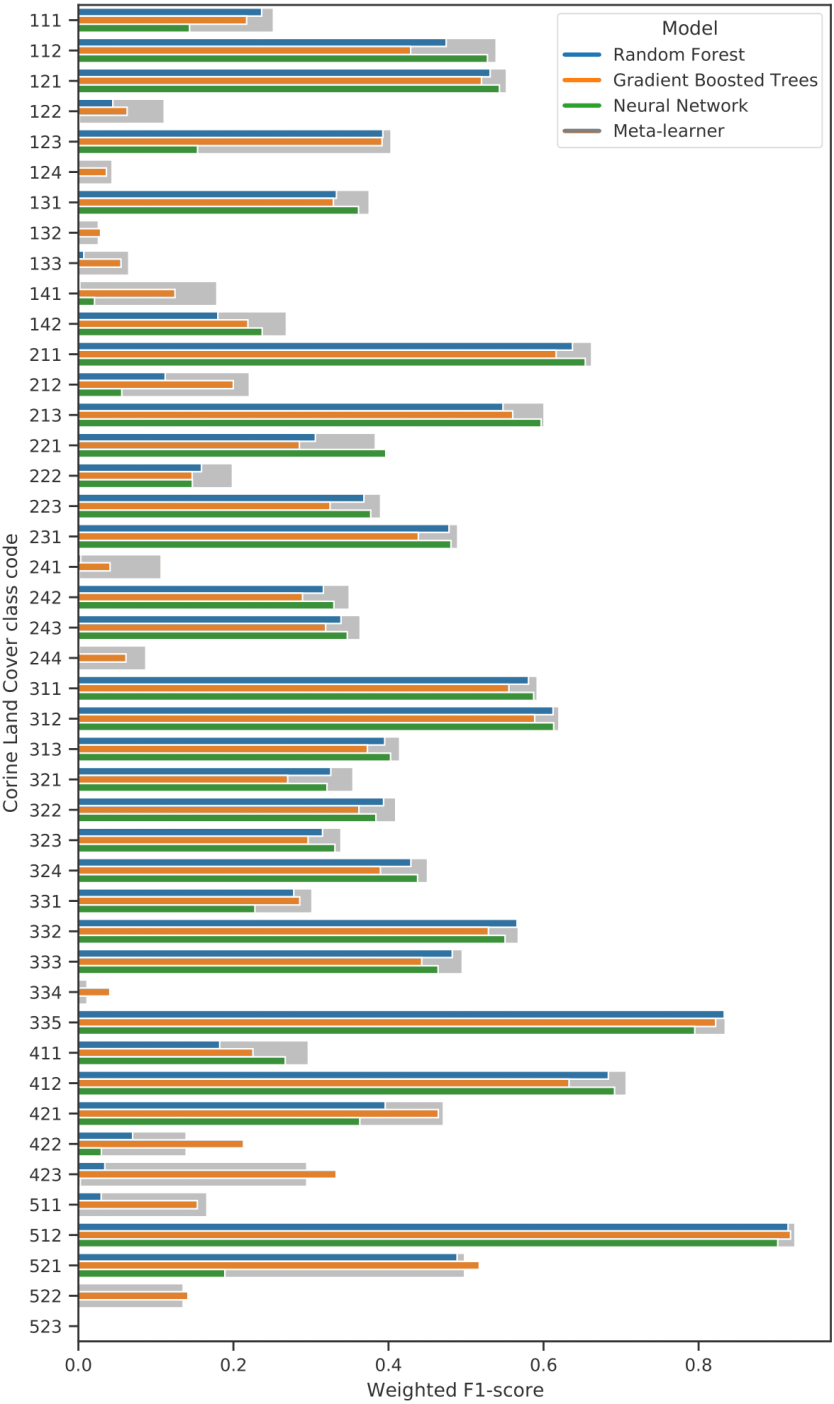
Grouped bar plot of the F1-scores CLC class, plotted separately per model of the ensemble. Meta-learner performance is indicated in red on the background of each bar. If the random forest (blue), gradient boosted trees (orange) or neural network (green) outperformed the meta-learner, its bar will exceed the bigger meta-learner bar, indicating that the meta-learner did not learn to incorporate the model’s higher performance into its final prediction.

We compared the F1-score of each component model and the meta-learner. The neural network achieved the highest weighted F1-score of 0.514. The meta-learner scored 0.513, the random forest 0.506, the gradient boosted trees 0.471. Figure 12 shows the difference in performance per model per class. When scored per class, the meta-learner achieved the highest F1-score on 36 out of 43 classes, the random forest on 1 class (523), the gradient boosted trees on 6 classes (132,334,422,423,521,522), and the neural network on 1 class (221).

### Time-series analysis results

**Figure 13:**
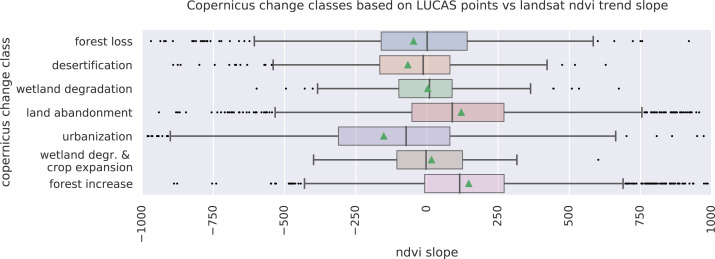
NDVI trend slope values of LUCAS points with selected LULC change dynamics, categorized according to the Copernicus change classes. The mean NDVI trend value is indicated with green triangles.

**Figure 14:**
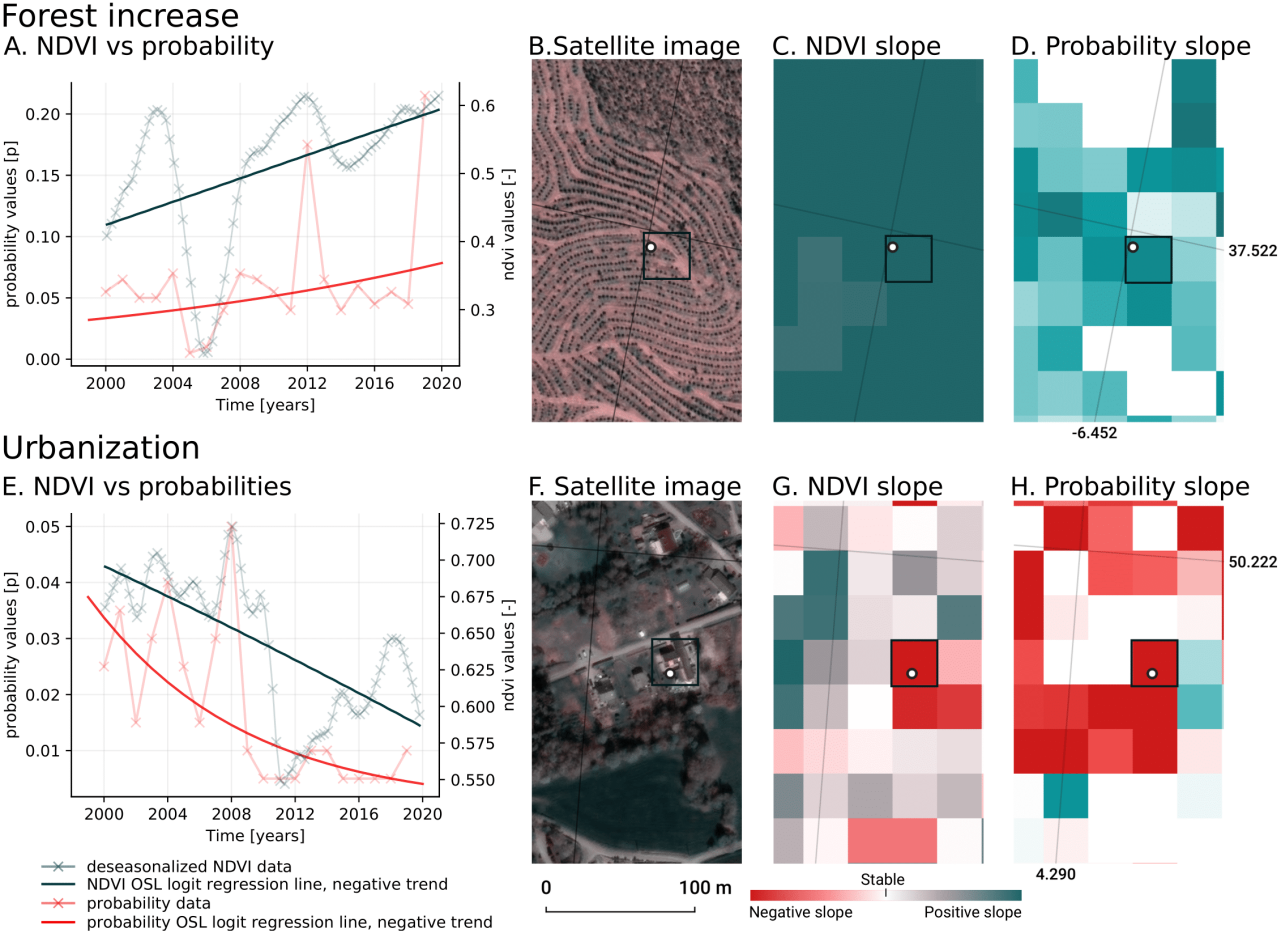
Detail plot of NDVI and LULC trends between 2000–2020 for 2 LUCAS points. NDVI trend is compared to forest increase (top) and urbanization (bottom). Left (A and E): A graph comparing the two trends, with green depicting de-seasonalized NDVI data and its trend, as calculated by logit OLS regression. Red depicts the annual probability values and associated trend of the compared LULC change classes (“312: Coniferous forest” and “111: Continuous urban fabric”, respectively). The maps, from left to right, depict the spatial context of the two points in (B/F) high-resolution satellite RGB, (C/G) slope of Landsat ARD NDVI trends, and (D/H) slope of LULC change class trends as predicted by our ensemble. The “in-situ” observations of both points match the dynamic presented in the graph: Point 28681762 (top) experienced forest increase, while point 39143028 (bottom) is located in a recently constructed urban area.

Our NDVI slope maps show which areas have an increase or decrease in NDVI over time. We selected 19500 LUCAS points that experienced LULC change and overlaid these with our NDVI slope values. Figures 13 and 14 show clear differences in NDVI trend between LUCAS points that have undergone different LULC change processes.

**Figure 15:**
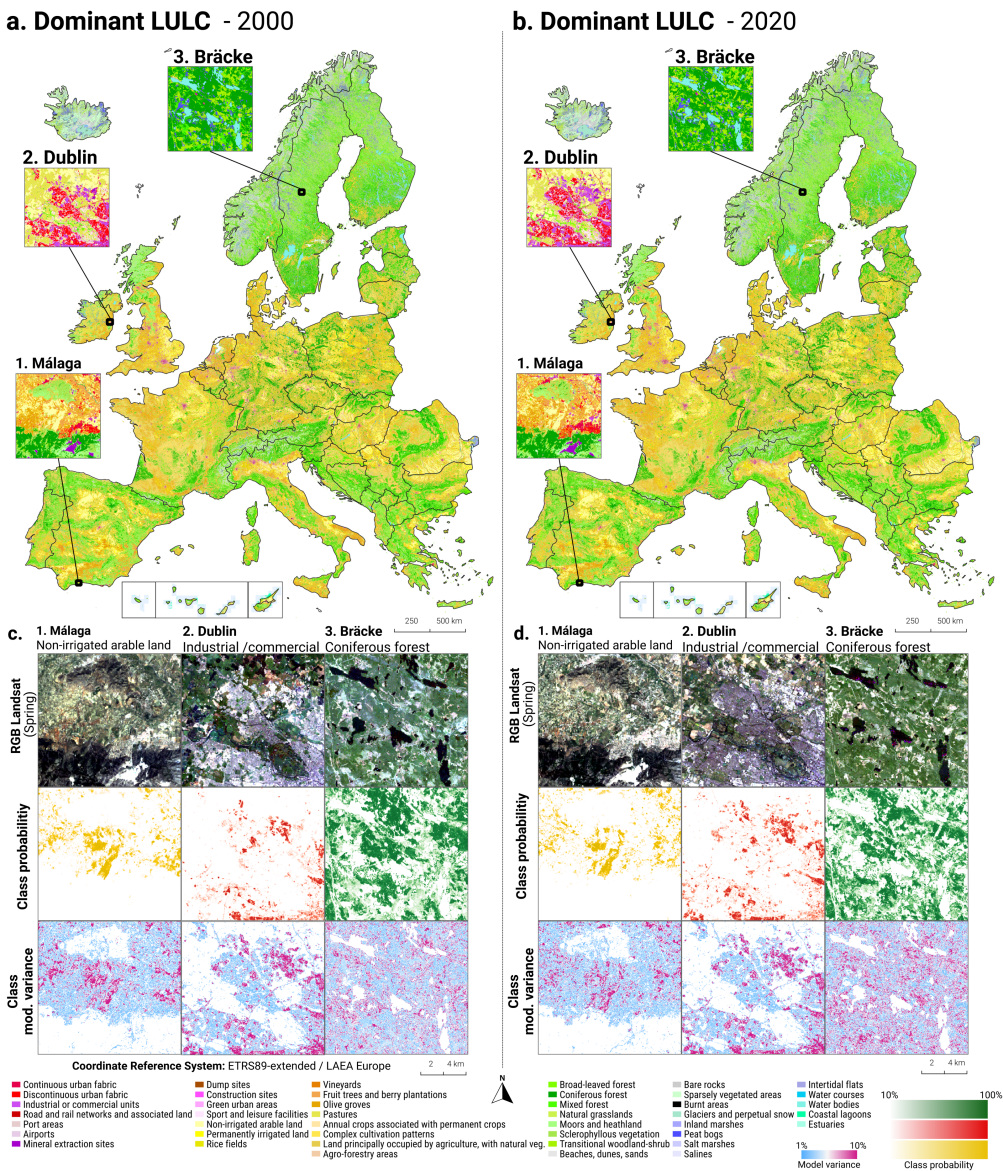
Dominant LULC classes, predicted probability and model variance for Non-irrigated arable land, Coniferous forest and Urban Fabric, RGB Landsat temporal composite (Spring season) for the years 2000 and 2019.

We generated annual maps for change classes (see Fig. 15 for the maps of 2000 and 2019). Filtered data as well as the removed noise can be viewed from the ODS-Europe viewer.

**Figure 16:**
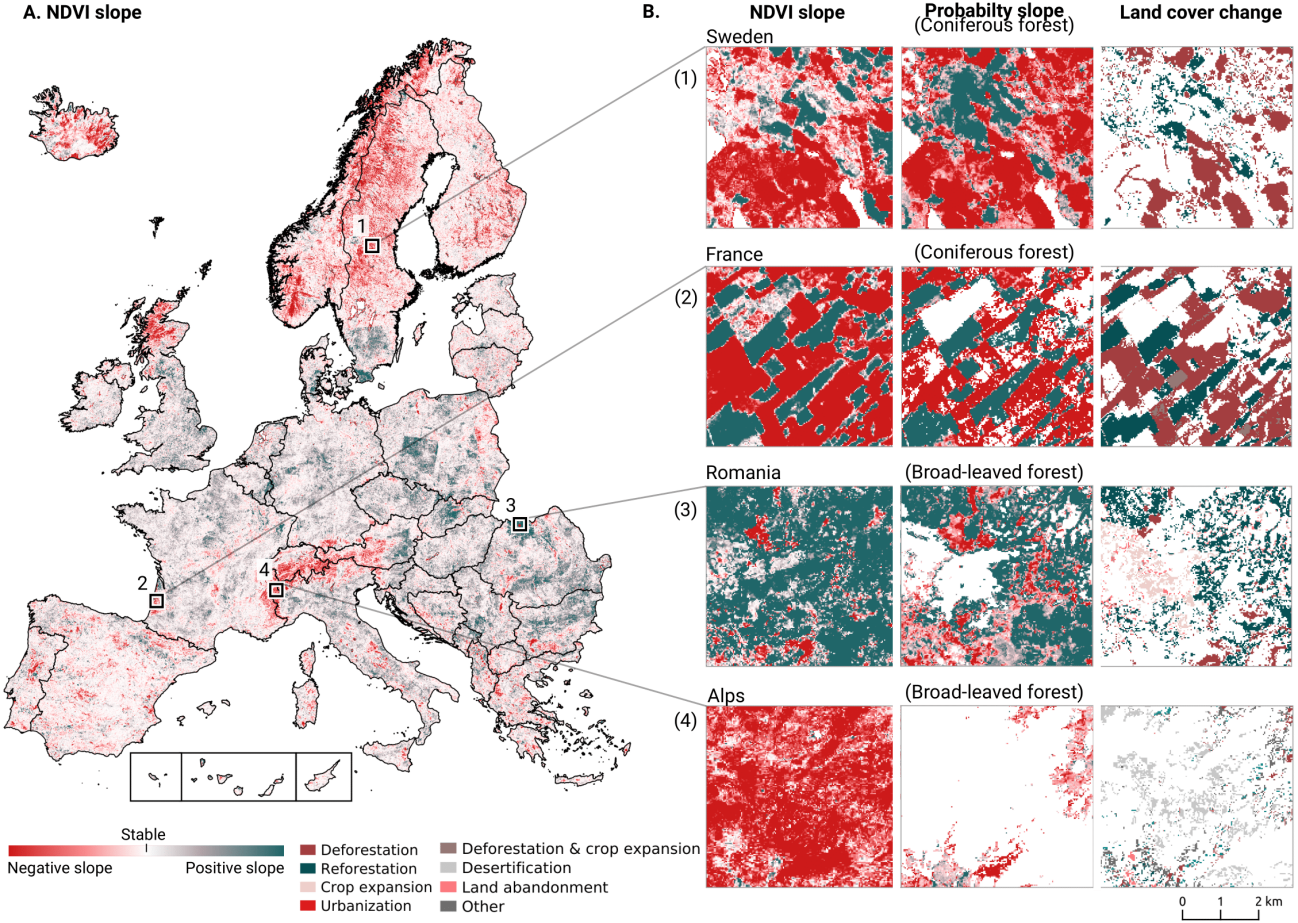
Trends in NDVI values between 2000 and 2019 compared to trends in LULC probabilities predicted by our ensemble model, as well as the derived LULC change classes between 2001 and 2018.

Figure 16 demonstrates how trend analysis can be used to explore large-scale trends and pixel-level details.

Figures 16B1 and 16B2 show areas of negative and positive slope occur adjacent to each other without gradual transitions. Figures 16B3 and 16B4 show examples of relatively large areas with homogeneous NDVI slope values. Overall, NDVI slopes in Europe tend to be positive, the largest exceptions being negative slope regions in Northern Scandinavia, Scotland, the Alps, South West France, Spain, Italy and Greece.

The right-most subplots of Fig. 16 show examples of where sudden land cover change classes at 30 × 30 m tend to match relatively large negative slopes, especially for change classes such as forest loss and urbanization.

**Figure 17:**
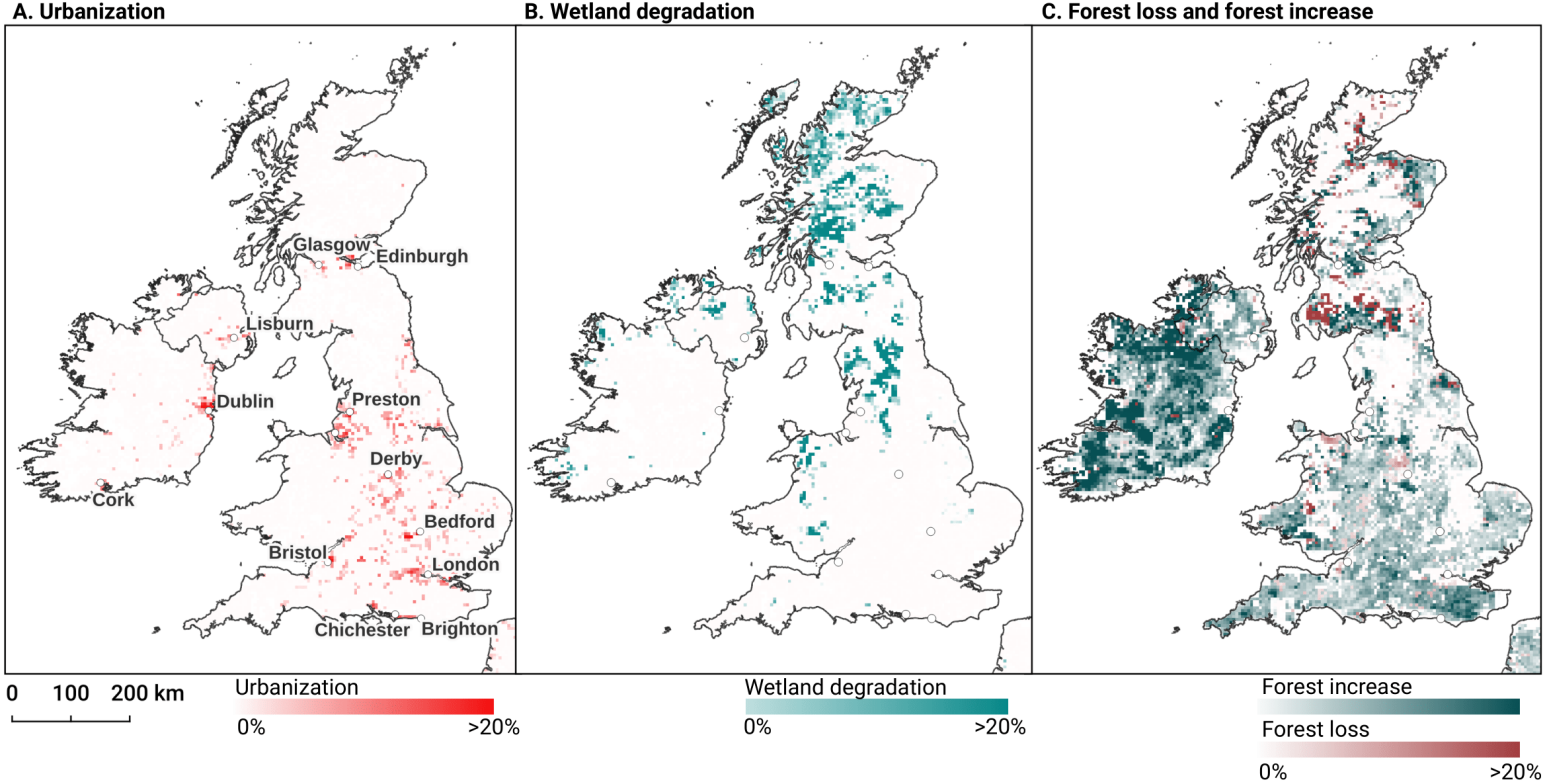
Prevalent LULC change and change intensity on the British isles aggregated to 5 × 5 km tiles, for three dynamics: Urbanization (A), Wetland degradation (B), and forest increase/decrease (C).

Figure 17 presents the long-term LULC change processes as suggested by our classification results. Figure 17A presents the dominant type of LULC change in a 5 × 5 km grid, while Fig. 17B shows the intensity of change as part of the total area on a separate map using 20 × 20 km areas. Large parts of mainland Europe are characterized with reforestation as the main change with patches of urbanization scattered in between. Norway, Sweden and Finland are characterized with forest loss as the main LULC change class. Large areas in Spain have land abandonment and crop expansion as the main land use class. When taking into account the intensity of the changes the central European countries seem to be stable with the Iberian peninsula, Scandinavia and parts of eastern Europe exhibiting more intense changes.

## Discussion

*“The appropriateness and adequacy of the 10-class schema used to describe land cover in today’s human-dominated world needs a serious rethink. What is the value of a 10 m (resolution) landcover map that cannot capture a grassland being turned into a solar farm?”*

**Mysore Doreswamy Madhusudan**

### Summary findings

We have presented a framework for automated prediction of land cover / land use classes and change analysis based on spatiotemporal Ensemble Machine Learning and per-pixel trend analysis. In this framework, we focused not only on predicting the most probable class, but also on mapping each probability and associated model variance. We believe that such detailed information gives a more holistic view of the land cover and land use and allows any future users to derive their own specialized maps of certain classes using probability thresholds and uncertainty per pixel and class, and/or to incorporate it in further spatial modeling.

**Table 16:** Weighted F1-scores obtained by validating spatial and spatiotemporal models on data from known years and an unknown year (2018). Trained on CLC points, LUCAS points, and a combination of both.

Model	Training year	Points	Trained on CLC		Trained on LUCAS		Trained on CLC and LUCAS				
			Tested on raining year (s)	Tested on 2018	Tested on training year (s)	Tested on 2018	Tested on training year (s)	Tested on 2018
Spatial	2000	100,000			0.610	0.542	0.611	0.515			
Spatial	2006	100,000	0.595	0.437	0.604	0.563	0.587	0.534			
Spatial	2009	100,000	0.595	0.482			0.602	0.415			
Spatial	2012	100,000	0.559	0.476	0.611	0.574	0.565	0.529			
Spatial	Average	400,000	0.583	0.465	0.608	0.560	0.591	0.498			
Spatiotemporal	All	100,000	0.612	0.576	0.568	0.478	0.574	0.532			
Spatiotemporal	All	400,000	0.625	0.579	0.608	0.491	0.595	0.543			

 We show that in the context of reproducing the CLC legend, models trained on multi-year observations generalize better to unknown years than models trained on single-year observations, and that ensemble machine learning marginally outperforms single classifiers overall. Our accuracy assessment however indicates that several CLC classes remain hard to reproduce in the proposed workflow. The on-par performance on the S2GLC validation points, however, suggests that the framework is capable of generating accurate predictions for relatively detailed legends if they do not contain heterogeneous classes.

We further explained the time-series analysis framework for processing partial probabilities and NDVI values aiming at detection of significant spatiotemporal trends. We provide pixel-wise uncertainty measures (standard deviation of the slope / beta coefficient and R-square), which can also be used in any further spatial modeling. The whole framework, from hyper-parameter optimisation, fine-tuning, prediction and time-series analysis, is fully automated in the (eumap python package https://eumap.readthedocs.io/) and generates consistent results over time with quantified uncertainty, making it more cost-effective for future updates and additions.

### Model performance

Our spatial cross-validation accuracy assessment results indicate limited hard-class accuracy (Weighted F1-score of 0.494) at the highest classification level (43 classes) with several classes such as *124: Airports*, and *334: Burnt Areas* performing poorly, likely rendering them unfit for further use. However, a comparison of each class’ separate log loss score indicates that the model predicted each class more accurately than the baseline. For example, *522: Estuaries* was one of the least accurately predicted classes in the hard-class classification, but had a log loss ratio of 0.566. This means that probabilities were frequently correctly assigned to validation points in estuaries but overshadowed by other, more numerous classes (*e.g.*, *512: Water Bodies*), allowing a more accurate mapping of estuaries by adjusting the probability threshold for that specific class. Furthermore, our validation on the independent S2GLC dataset collected by Malinowski et al. (2020) indicates that the accuracy of our model is comparable to the model used in their publication. Our conservative estimate (counting all points with predicted classes outside the S2GLC legend as errors) resulted in a weighted average F1-score of 0.854 and a kappa score of 0.794 and our optimistic estimate (where those points were removed before calculation) yielded F1: 0.889 and kappa: 0.867, while Malinowski et al. (2020) reported 0.86 and 0.83, respectively. While these points were sampled to validate a 10 m resolution map and it is unclear how this affects the accuracy assessment, we could not find a reason to expect overestimated accuracy values in existing literature.

This suggests the nomenclature used by Malinowski et al. (2020) is more optimized for remote sensing-based classification than the CLC legend and that the framework presented in this work is capable of achieving accuracy levels comparable to state-of-the-art 10 m resolution land cover products when using a more suitable legend. However, when we transformed our cross-validation results to the S2GLC legend, we obtained an F1-score of 0.611 and a kappa score of 0.535, which is considerably lower. This is unlikely to happen when comparing two datasets that are both sampled in a representative, proportional approach; it is therefore likely that the mismatch is caused by the training points in the ODSE-LULC dataset that were generated from CLC centroids.

The average weighted F1-score per year was 0.489 with a standard deviation of 0.135, while the average weighted F1-score per tile was 0.463, with a standard deviation of 0.150. This means that our model was more consistent through time than through space. A possible explanation is the unequal distribution of training points derived from the CLC data; we did not sample this data based on how much area they cover, but instead on how many separate areas occur in the data. Regions of Europe and classes with smaller CLC polygons may be over-represented in the data. Fig. 8 shows that there is a slight but significant correlation between the number of points and cross-validation F1-score. This suggests that improving the CLC sampling strategy may improve the spatial consistency of our model.

### Advantages and limitations of combining CLC and LUCAS points

We included LUCAS points in our dataset in order to base our modeling and predictions on a consistent and quality-controlled dataset. However, in this work we found that training spatiotemporal models on LUCAS points lead to lower classification accuracy estimates than when only using CLC points (see Table 16). This was unexpected, as LUCAS land cover information stems from actual ground observations, while the CLC points are pseudo-ground truth points from a dataset with a large minimum mapping unit. This suggests that either the LUCAS points are harder to reproduce with remote sensing techniques, or that the harmonization and data filtering process needs to be improved. Further testing is needed to clarify this.

### Advantages and limitations of using spatiotemporal models

The results of testing the generalization potential of spatiotemporal models with separate experiments (see methods and results sections about spatial *vs* spatiotemporal machine learning) show that spatiotemporal models generalize better to data from years they were not trained on. These findings suggest that we can use the existing model to predict land cover for 2020 and 2021 without collecting new training data: Preparing Landsat images for these periods would be likely enough.

Our results also suggests that we can use contemporary reference data to make consistent predictions for periods *prior* to the year 2000, for which very little training data is available. We intend to produce predictions for the years 1995, 1990 and to 1985 in the next phase of our project. We did not do this previously because the Landsat ARD data (Potapov et al., 2020) is only available after 1997. We need to compute and re-calibrate the Landsat 5, 6 and 7 products ourselves, which adds a higher level complexity due to the differences in sensors and acquisition plans.

Another limitation for this work is the fact that the long-term spatiotemporal approach aims at 30 m resolution data, while most current land cover products aim at a 10 m resolution. Furthermore, our approach is highly dependent on the availability of quality reference data from multiple years. Many continents except North America and Australia do not have access to datasets similar to LUCAS, which might become real challenge for applying the framework outside Europe, and especially in Africa, Latin America and Asia.

### Advantages and limitations of using ensemble models

We implemented ensemble machine learning in our framework for two main reasons. Firstly, to achieve the highest accuracy possible, and secondly, to allow for the inclusion of model variance as a proxy for the uncertainty of its predictions (Zhang & Ma, 2012). Our results indicate that using an ensemble approach can indeed increase accuracy. Although the neural network component model scored a slightly higher weighted average F1-score than the meta-learner, the meta-learner achieved the highest F1-score on most classes, suggesting that the meta-learner sacrificed a slight amount of overall performance in order to improve performance on classes that the neural network could not recognize.

**Figure 18:**
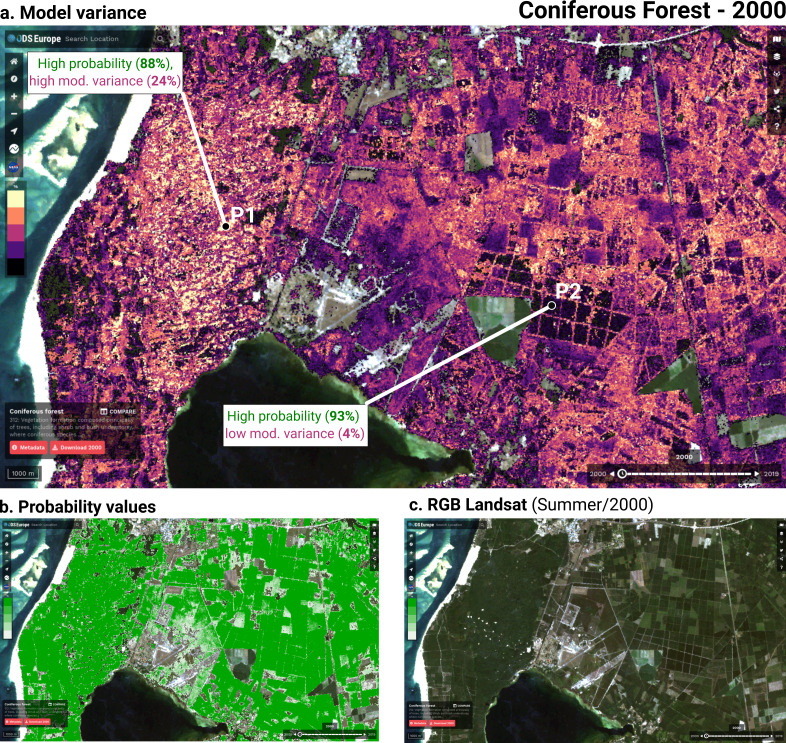
Example of model variance (prediction uncertainty) in he city is of La Teste-de-Buch (France) for the class “Coniferous forest”, visualized in the ODSE viewer (https://maps.opendatascience.eu/). (A) model variance map with examples of two locations (P1 in 44°33′33.6′N 1°10′33.2′W; P2 in 44°32′11.8′N 1°02′38.0′W) with low and high variances, (B) probability values showing relatively high confidence, (C) original Landsat images RGB composite used for classification.

Another advantage of doing ensembles with 5–fold CV with refitting of models and then stacking, is that we can generate maps of model variance (showing where multiple models have difficulties predicting probabilities). This allows users to identify problem areas (see Fig. 18), determine where best to collect additional samples, or adjust their classification legend or probability thresholds. To our knowledge, mapping model error of predicted probabilities is a novel area and none of existing landcover datasets for EU provides such information on a per-pixel basis.

### Time-series analysis, interpretations and challenges

**Figure 19:**
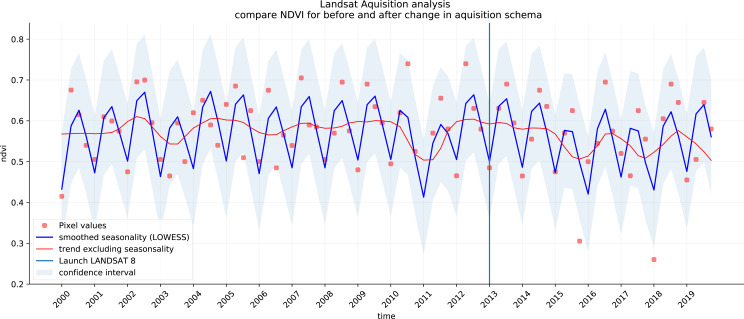
NDVI signal for 880 million pixel values in our Landsat data between 2000 and 2019. Red dots indicate the average for each season for 880 million pixels over 11 tiles. The vertical line indicates the launch of Landsat 8, after which the acquisition scheme changed. This sample suggests that the structural difference between the two acquisition schemes in the Landsat ARD product created by Potapov et al. (2020) were not propagated into our aggregated and harmonized dataset.

Palahi et al. (2021) found that the transition between Landsat 7 and 8 caused temporal inconsistency in the reflectance data. We tested whether these inconsistencies were propagated into our aggregated and harmonized dataset by calculating the NDVI values of 11 million pixels of our dataset. We then performed a two-sided *t* test in order to analyze whether there was a difference in NDVI values before and after the launch of Landsat 8 in 2013 (see Fig. 19). The *t* test did not indicate a significant difference (test statistic of 0.0 and *p* = 1.0) between the two distributions, suggesting that the inconsistencies from the transition were not propagated through our preprocessing step.

The results of the probability trend analysis show some interesting patterns. We have focused on four geographic areas: (1) Sweden, as its forest dynamics have already garnered academic attention and it is an exemplary area where remote sensing techniques and on the ground measurements might come to different conclusions (see e.g. Ceccherini et al. (2020)). (2) South West France, as it is similar to the Sweden both in our data and is also compared by other authors (Senf & Seidl, 2021). (3) Northern Romania because it shows a large region with positive trends for both NDVI and broad-leaved forest land cover, suggesting it is reforesting at high rates. Finally, we found large regions in the Alps (4) that show a strong negative trend for NDVI values that does not seem to correspond to a clear land use change. This signal in our data suggests there may be more artifacts and that further research is needed.

Forest loss in Europe is currently highly debated in academia (Senf et al., 2018; Ceccherini et al., 2020; Senf & Seidl, 2021; Palahi et al., 2021; Picard et al., 2021). Discrepancies between national forest inventories and remote sensing techniques has led to disagreements in Sweden (Paulsson et al., 2020), Finland (Breidenbach et al., 2020), and Norway (Rossi et al., 2019). For instance, it was found that existing remote sensing products are deemed not fit for these types of analysis (Palahi et al., 2021). For these reasons, and because we do not validate our trend results, we neither attribute specific causes, nor do we analyze differences between specific time periods.

Further comparison of the most prominent change between 2001–2018 and our results suggest that forest is disappearing more than it is re-appearing in multiple locations. This is corroborated by Global Forest Watch forest gain data; for example, the Jämtland region in Sweden lost 287 k ha of tree cover and gained 164 k ha between 2001 and 2012 (Hansen et al., 2013). We present the case of the Landes region in France here as well as it shows a similar pattern to large parts of Sweden and is a known area for large scale forest harvesting (Senf & Seidl, 2021). These cases exemplify the usefulness of our maps for finding similar processes all over Europe by using a combination of the data that is presented here. More testing and ground-validation of the land cover changes is needed to assess which changes are over-estimations and which are realistic.

Our data suggests that reforestation is the most prominent land cover change dynamic on a European scale. This change is accompanied by an observed increase of NDVI values. This observation is corroborated by the FAO’s State of Europe’s Forests report 2020 which states that European forest cover has increased by 9% between 1990 and 2020 (Raši, 2020) and with global estimates that forest cover has increased by 7% between 1982 and 2016 (Song et al., 2018). This increase is consistent with expectations that increased CO2 will enhance plant growth in general. Another concern that is raised is that most of the increase in forest gain is by planted forests (Payn et al., 2015) that are less valuable in terms of biodiversity and carbon sequestration (Liu et al., 2018) and less adaptable to climate change. One exemplary area with observed reforestation is found in Northern Romania in all parts of our time-series analysis: we see a change from grassland to forests making reforestation the dominant change class, the broad-leaved forest class probability is increasing, and NDVI values show positive trends.

Finally, our data shows unexpected negative NDVI trends for large parts of the Alps. This may be related to changes in snow cover as found by Wang et al. (2018) in the Tibetan Plateau and by Buus-Hinkler et al. (2006) in the Arctic regions. However, this is not corroborated by the probability slope for class *“Glaciers and perpetual snow”* in our data. It is also possible that this is an artifact from our gap-filling step. Again, further study is necessary before any conclusions can be drawn.

### Future work

Even though our framework is comprehensive and has produced predictions of comparable accuracy to the current state-of-the-art on a less complex legend (see results section on S2GLC), after almost 14 months of processing the data and modeling land cover, we have found that that many aspects of our system could be improved:

• *Improving performance without sacrificing detail*: We consider the poor performance on the 43-class level 3 CLC legend to be the main weakness of our approach. Including such a large and hierarchical legend theoretically makes the resulting data more useful to more potential users, but this will only manifest if the classifications are also reliable for research and policy. To this purpose, we will continue research on methods to improve classification performance while maintaining (or expanding) thematic resolution.
• *Cross-validation of land cover trends*: It was beyond the scope of our project to validate the results of our long-term trend analysis. Independently identifying and quantifying both sudden land cover changes (*e.g.*, due to natural hazards such as fires and floods) and gradual dynamics such as urbanisation and vegetation succession. We have however published all our data online, enabling other research groups to test their usability for land monitoring projects.
• *Combining classification with Object-Based Image Analysis (OBIA) and pattern recognition*: Incorporating spatial context to our workflow could potentially improve performance for several classes that are defined by land use. For instance, class 124: *“Airports”* was frequently misclassified as either urban fabric, non-irrigated arable land, pastures, or Sport and leisure facilities, another complex class that contains buildings and green areas. These predictions likely matched the land cover of the pixel, but missed the spatial patterns that make airports easily recognizable by humans (elongated landing paths). The same issue applies to most other artificial surface LULC classes. The relatively high importance of the TRI of the Landsat green band (see Fig. 7) suggests that additional feature engineering or other forms of incorporating the spatial context would improve classification performance on complex classes.

The field of land cover mapping is rapidly evolving. With exciting new global 10 m resolution products such as ESA WorldCover and Google’s Dynamic World Map expected in 2022, we expect the LULC mapping bar to be raised quickly to higher resolution and higher accuracy. Venter & Sydenham (2021) used low-cost infrastructure to produce land cover map of Europe at 10 m—thanks to ESA and NASA making the majority of multispectral products publicly available, today everyone could potentially map the world’s land cover from their laptop. Szantoi et al. (2020) show that many land cover products, however, are often ill-suited for practical actions or policy-making. As the quote at the start of this sections says *“The appropriateness and adequacy of the 10-class schema used to describe land cover in today’s human-dominated world needs a serious rethink”*, we assert that one should not look for land cover classification legends that are *“low-hanging fruits”* for the newest Sentinel imagery, but build people- and policy-oriented datasets that can directly help with spatial planning and land restoration. Our primary focus, thus, will remain on producing harmonised, complete, consistent, current and rapidly-updatable land cover maps that link to the past and allow for the unbiased estimation of long-term trends. We intend for this type of data to facilitate a better understanding of the key drivers of land degradation and restoration, so that we can help stakeholders on the ground make better decisions, and hopefully receive financial support for the ecosystem services our environment provides to us all.

## Conclusion

The spatiotemporal ensemble machine learning framework presented achieved a cross-validation weighted F1-score of 0.49, 0.63, and 0.83 when predicting 43 (level-3), 14 (level-2), and 5 classes (level-1). These values are lower than those reported by other current works that use classification systems with more optimized legends, and less classes. Our validation on an independent test dataset (Malinowski et al., 2020) with such an optimized legend yielded accuracy metrics comparable to Malinowski et al. (2020). This indicates that the framework is capable of achieving similar performance to state-of-the-art methods, without any post-processing, and on a coarser spatial resolution, given a less ambitious task.

In our experiments, spatiotemporal models generalized better to EO data from previously unseen years: Spatiotemporal models outperformed spatial models on known-year classification by 2.7% and unknown-year classification by 3.5%. This suggests that spatiotemporal modeling, as incorporated in the presented framework, can be used to predict LULC for years of which no LULC observations exist, even prior to 2000 and beyond 2020.

Other methodological advantages of using spatiotemporal ML are (1) that it helps produce harmonized predictions over the span of years, (2) that the fitted model can be used to predict LULC in years that were not included in its training dataset, allowing generalization to past and future periods, *e.g.* to predict LULC for years prior to 2000 and beyond 2020. Also, it is an inherently simple system with whole land cover of EU represented basically with a single ensemble ML (a single file). The disadvantages of using spatiotemporal ML is that it requires enough training points spread through time, and EO data needs to be harmonized and gap-filled for the time-period of interest (in this case 2000–2019). Also, it is computationally at the order of magnitude more complex than spatial-only methods. Producing uncertainty per pixel for each class significantly increases data volume and production costs.

Time-series analysis of predicted LULC probabilities and harmonized NDVI images over continental Europe suggests forest loss in large parts of Sweden, the Alps, and Scotland. The Landsat ARD NDVI trend analysis in general matches the land degradation/reforestation classes with urbanization resulting in the biggest decrease of NDVI in Europe.

## Additional Information and Declarations

### Data Availability

The following information was supplied regarding data availability: The harmonized training samples are available at Zenodo: Martin Landa, Lukas Brodsky, Leandro Parente, Martijn Witjes, & Tomislav Hengl. (2021). Multi-year harmonized land cover samples based on LUCAS and CORINE datasets (v0.1) [Data set]. Zenodo. https://doi.org/10.5281/zenodo.4740691. Dominant lc classes and spatiotemporal model (LandMapper instance) are available at Zenodo: http://doi.org/10.5281/zenodo.4725429
Leandro Parente, Martijn Witjes, Tomislav Hengl, Martin Landa, & Lukas Brodsky. (2021). The continental Europe land cover mapping at 30m resolution based CORINE and LUCAS on samples (v0.1) [Data set]. Zenodo. https://doi.org/10.5281/zenodo.4725429. The continental EU DTM are available at Zenodo: Hengl, Tomislav, Leal Parente, Leandro, Krizan, Josip, & Bonannella, Carmelo. (2020). Continental Europe Digital Terrain Model at 30 m resolution based on GEDI, ICESat-2, AW3D, GLO-30, EUDEM, MERIT DEM and background layers (v0.3) [Data set]. Zenodo. https://doi.org/10.5281/zenodo.4724549
Most of the layers available via the ODS-Europe viewer (https://maps.opendatascience.eu) are also available seamlessly through our S3 Cloud Object Service as Cloud-Optimized GeoTIFFs under the Open Data Commons Open Database License (ODbL) and/or Creative Commons Attribution-ShareAlike 4.0 and/or Creative Commons Attribution 4.0 International license (CC BY).
